# The “Cocombola Study”: A Physical Phantom Model for tDCS-Induced Electric Field Distribution

**DOI:** 10.3390/bioengineering12040346

**Published:** 2025-03-27

**Authors:** Matteo Guidetti, Rosanna Ferrara, Kora Montemagno, Natale Vincenzo Maiorana, Tommaso Bocci, Sara Marceglia, Serena Oliveri, Anna Maria Bianchi, Alberto Priori

**Affiliations:** 1 ‘Aldo Ravelli’ Research Center for Neurotechnology and Experimental Neurotherapeutics, Department of Health Sciences, University of Milan, 20142 Milan, Italy; matteo.guidetti@unimi.it (M.G.); roferrara@unisa.it (R.F.); kora.montemagno@hotmail.com (K.M.); natale.maiorana@unimi.it (N.V.M.); tommaso.bocci@unimi.it (T.B.); sara.marceglia@unimi.it (S.M.); serena.oliveri@unimi.it (S.O.); alberto.priori@unimi.it (A.P.); 2 Department of Electronics, Information and Bioengineering, Politecnico di Milano, Piazza Leonardo da Vinci, 32, 20133 Milan, Italy; 3 III Neurology Clinic, ASST-Santi Paolo e Carlo University Hospital, 20142 Milan, Italy

**Keywords:** transcranial direct current stimulation, tDCS, modeling, phantom head model

## Abstract

Background: Transcranial direct current stimulation (tDCS)-induced electric fields (EFs) acting on brain tissues are hardly controllable. Among physical models used in neuroscience research, watermelons are known as head-like phantoms for their dielectric properties. In this study, we aimed to define an inexpensive and reliable method to qualitatively define the spatial distribution of tDCS-induced EFs based on the use of watermelons. Methods: After creating the eight cranial foramina and identifying the location of the 21 EEG scalp electrodes on the peel of a watermelon, voltage differences during stimulation were recorded in each of the 21 scalp electrode positions, one at a time, at four different depths. The recordings were graphically represented by using polar coordinates with the watermelon approximated to a perfect sphere. Results: To validate the model, we performed three experiments in well-known montages. The results obtained were in line with the expected behavior of the EFs. Conclusions: Watermelon might be a cheap and feasible phantom head model to characterize the EFs induced by tDCS and, potentially, even other non-invasive brain stimulation techniques.

## 1. Introduction

Transcranial direct current stimulation (tDCS) is a non-invasive brain stimulation technique that modulates brain activity through the injection of a low-intensity direct current (2–4 mA) across the scalp [1,2]. Although decades of studies have explored a wide range of clinical applications, both in healthy and pathologic subjects (for a review, see ref. [3]), showing beneficial effects with no harmful side effects [4], it is still hard to determine the treatment efficacy [5,6]. Indeed, tDCS-induced biological and behavioral effects depend upon individual anatomy (considered in a broad sense [7,8,9,10,11]) and the small electric field (EF) developed in the brain [12]. Also, tDCS-induced EF is hardly controllable [13]. Physically, the intensity and distribution of tDCS-induced EFs depend upon the characteristics of the current injected, namely temporal (e.g., waveform) and spatial (e.g., electrodes’ position) [12]. Therefore, a large number of stimulation variables play a role, and it remains a big challenge to select stimulation parameters for the best medical practices [6,14].

Computational simulations have been applied to determine the spatial–temporal distributions of the EFs within the brain [15], providing insights into the stimulation parameters to be chosen [6]. MRI-based realistic human models that incorporate complex tissue geometries [16] with dielectric properties [17,18] are currently considered the gold-standard tools [19,20]. While useful, they have limitations [21], including the accuracy and precision of MRI tissue segmentation [22], the assignment of tissue conductivities [23,24], and the numerical artifacts introduced by stair-casing error [25]. Therefore, computational predictions must be experimentally validated before a direct translation to clinical practice [6,14,21]. In the last few years, several models have been used to confirm the computational results and, in general, shape tDCS-induced EFs. Few studies have studied humans undergoing neurosurgery to record EFs induced by tDCS [26]. Although ideally in vivo recordings would be the most reliable [22], in practice, there are several factors that might mislead the recordings and interpretation—for example, the anatomical and pathophysiological characteristics of the subjects, or the technical and methodological aspects of the recording [26]. Another approach to model tDCS-induced EFs is exploiting phantom head models, i.e., models reflecting human brain biophysics (e.g., conductivity) and anatomy (e.g., weight, shape) [14], using materials with different conductivity (e.g., ceramics, clay, plastic) [27,28]. Compared to computational simulations, phantoms consider real-world influences, such as environmental noise or 3D positioning errors [28]; compared to in vivo recordings, physiological uncertainties (e.g., personal neurophysiological features) do not exist [28] and experiments are repeatable multiple times [14]. Also, phantom head models allow for real-time recordings and training in different settings [21].

Melons and watermelons have been used as head-like phantoms to improve neurophysiological [29,30,31], neurostimulation [30,32], and neuroimaging [31,33] practices. For example, watermelon as a phantom head model has been used as a control condition to identify and assess stimulus-related artifacts when recording EEG during transcranial magnetic stimulation (TMS) [29] and tDCS [34]. Similarly, it was used to ensure safety and quantify tDCS-induced noise effects in the images while performing tDCS concurrent with fMRI [33]. From a bioelectrical point of view, melon and watermelon share similar dielectric properties (or permittivity) [35], which might be a reasonable approximation of the human head. The dielectric constant of their peel is comparable to human skin, being, respectively, ε0 = 30–50 (frequency range 200 MHz–2 GHz, 24 °C—[36]) and ε0 = 40–50 (frequency range 200–500 MHz, 36 °C—[37]), and their inner pulp has a higher dielectric constant than their peel [36]. A similar physical trend is found in the human head, with gray matter and muscle in comparison to human skin [38]. Nevertheless, no previous tDCS modeling studies have been conducted on watermelon.

Under the hypothesis that watermelons may represent a non-expensive and reliable physical phantom head model for tDCS, here, we propose a method to use watermelons to qualitatively characterize the spatial distribution of tDCS-induced EF, together with an example of practical application described in the Supplementary Materials.

## 2. Materials and Methods

For modeling the spatial distribution of tDCS-induced EF, we propose to use a watermelon (*Citrullus lanatus*) whose shape (circumference around 60 cm [39]) and weight (around 4 kg [40]) are similar to the average human head. Detailed descriptions of the validation procedures are reported in the Supplementary Materials.

### 2.1. Watermelon Preparation

**Figure 1:**
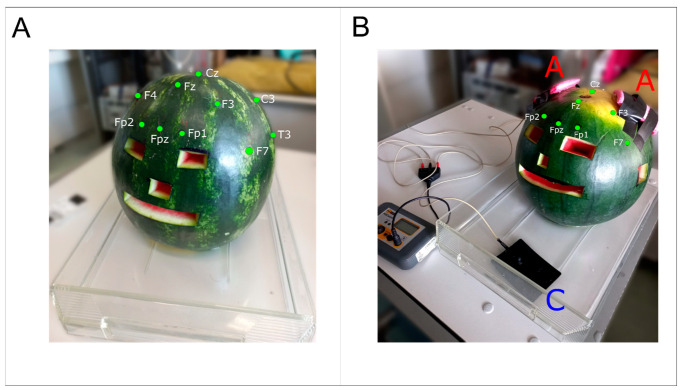
(**A**) Watermelon with 10–20 system in plastic box with water. (**B**) Watermelon in CONDITION A. A = anode, C = cathode.

Experiments were performed in one day, to prevent fruit decay. After taking the watermelon from a room where it was stored at 24 °C, we identified the location of 21 scalp electrodes according to 10–20 conventional systems [41] to ensure a homogenous coordinate system during stimulation and recording procedures and facilitated the results description. The four anatomical landmarks used for positioning the electrodes (nasion, inion, right and left preauricular zones) were chosen by taking real measurements from a healthy volunteer. Similarly, we made eight holes (orbital, nasal, oral, ear cavities, and cranial foramen magnum) in the peel to simulate cranial foramina according to proportions taken from the same human model. Before starting the stimulation session, the weight (kg), maximum diameter (mm), temperature (°C), and resistance (kΩ) at the peel and the pulp were taken (see Supplementary Materials, Table S1). The watermelon was placed in a plastic box to electrically isolate the phantom and immersed in a few millimeters of water to simulate the electric dispersion induced by cerebrospinal fluid [42] (Figure 1A).

### 2.2. Recording Set Up

The signal was recorded from two self-adhesive bipolar surface Ag–AgCl electrodes (15 × 20 mm) connected to a digital oscilloscope (Tektronix TDS2024C oscilloscope—Tektronix, Inc., Johnston, IA, USA): one electrode (1.5 × 2 cm) was attached directly to the stimulating surface of tDCS electrode and considered as the reference for recording, and the other (1.5 × 2 cm) was wrapped around one end of a 48.5 cm-long and 5 mm-wide copper round bar (contact surface: 98.12 mm^2^), accurately covered with insulating tape. The other end of the copper wire was used as the recording area (98.12 mm^2^). The recording electrode was inserted in the watermelon in each of the 21 scalp electrode positions one at a time, at four depths: on the surface (Z_0_, roughly resembling human skin), at 33% (Z_33_, roughly resembling human cortex), at 66% (Z_66_, roughly resembling human diencephalon and midbrain), and at 100% (Z_100_, roughly resembling human brainstem) of the diameter of the watermelon. During stimulation, we recorded the voltage difference (ΔV) between the two recording electrodes, and EF as ΔV/Δs (Δs = distance between each recording point and the origin of the reference system). After recording, each hole was filled with saline solution [27]. No amplification, band-pass filters, or ameliorative acquisition procedures were applied. In all conditions, signal acquisition occurred when the DC stimulation intensity was maximal and steady.

### 2.3. Graphical Representation

**Figure 2:**
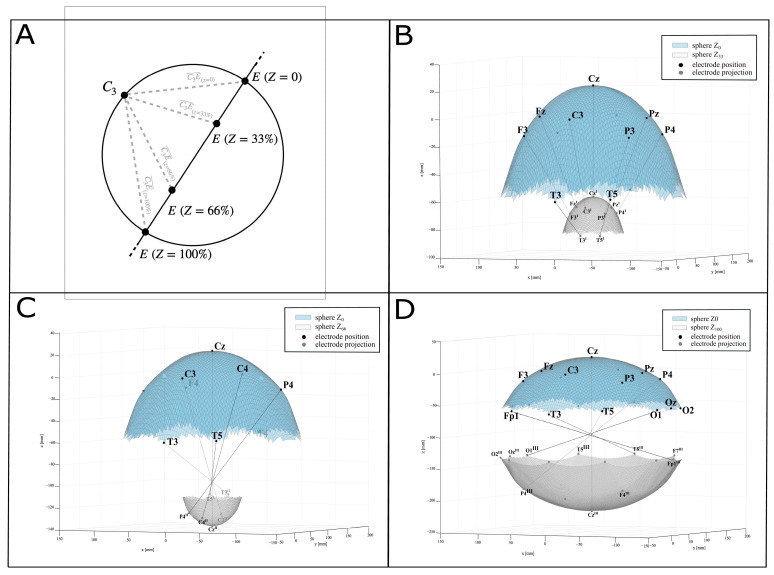
(**A**) Approximation to a perfect sphere. Example of calculation of distance between each recording point E (E_x_, E_y_, E_z_) at each depth Z and the origin of the reference system R. (**B**–**D**) Projection of 10–20 system recording point from the sphere Z_0_ to the spheres Z_33_, Z_66_, and Z_100_.

To represent the results in a simple and comparable graph, we approximated the watermelon to a perfect sphere, as previously reported [43]. We calculated the 3D coordinates of each recording point E (E_x_, E_y_, E_z_) at Z_0_, Z_33_, Z_66_, and Z_100_ by using polar coordinates (ρ, φ, θ). Then, we translated the obtained sphere model into the sphere model centered in the references for the recording system R (R_x_, R_y_, R_z_). Finally, we calculated the distance between each recording point E (E_x_, E_y_, E_z_) at each depth Z, and the references for the recording system R (R_x_, R_y_, R_z_), i.e., the distance RE (see Figure 2A). EF was calculated as the ratio between the experimentally recorded ΔV (mV) and the inferred distances RE (mm). We represented ΔV and EF at each of the four recording depths as four perfect concentric spheres (Z_0_, Z_33_, Z_66_, Z_100_), on which we projected the 10–20 points from Z_0_ where the points were marked (see Figure 2B–D). To note, we could not register ΔV in the recording points under the stimulating pads; therefore, for these values at Z_0_, Z_33_, Z_66_, and Z_100_, we averaged the surrounding ΔV at each depth. More details are available in the Supplementary Materials.

### 2.4. Validation

**Figure 3:**
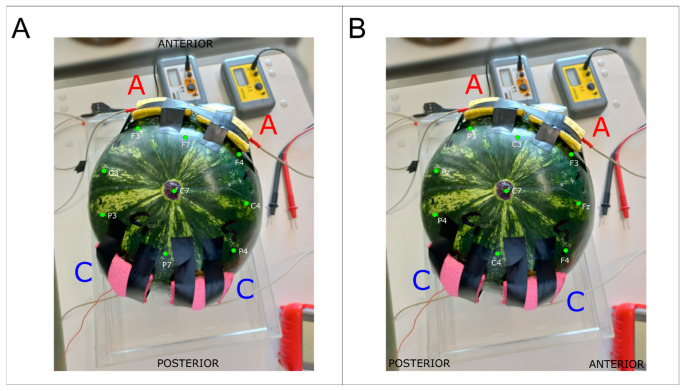
(**A**) Watermelon in EXPERIMENTAL CONDITION B. (**B**) Watermelon in EXPERIMENTAL CONDITION C. A = anode; C = cathode.

Model validation for tDCS-induced EF distribution cannot be performed against a golden standard because each modeling strategy has limitations, and none can be used as a reference. Therefore, to evaluate the validity of the model, we relied on the testing of the method performance in known situations and assessed whether the results obtained were in line with the expected results in terms of (I) EF distribution with respect to stimulating electrode position, (II) EF gradient from surface to the internal locations, and (III) the amount of current shunted through the surface. To do so, we tested our methodology using three tDCS montages: (1) CONDITION A: monopolar montage—two anodes over simulated motor cortices (7 × 5 cm^2^ over C_3_ and C_4_), reference over simulated right deltoid (8 × 6 cm^2^ in the water) (see Figure 1B); (2) CONDITION B: bicephalic fronto–occipital montage—two anodes over simulated prefrontal cortex (5 × 5 cm^2^ over Fp_1_ and Fp_2_), two references over simulated occipital cortex (5 × 5 cm^2^ over O_1_ and O_2_) (see Figure 3A); and (3) CONDITION C: bicephalic fronto–temporal, two anodes over simulated left ventrolateral prefrontal and occipitotemporal cortex (5 × 5 cm^2^ over F_7_ and T_6_), references over simulated right ventrolateral prefrontal and occipitotemporal cortex (5 × 5 cm^2^ over F_8_ and T_6_) (see Figure 3B). DC stimulation was applied using a stimulator (2 mA, CONDITION A) or two stimulators (1.4 mA, CONDITIONs B and C) (HDCStim, Newronika, Cologno Monzese, Italy) through silicone rubber pad electrodes (1 mm thickness), with conductive gel applied between the electrodes and the peel to lower tDCS electrode resistance. For each stimulation condition, we used a watermelon (n = 3) whose shape and weight were similar to the average human head, considering that dielectric properties remain constant among them [36]. Also, we kept the temperature of the watermelon constant at around 24 °C [44] throughout the duration of the experiments.

## 3. Results

As for the purpose of this study, EF was measured as the ratio between the experimentally recorded ΔV (mV) and the inferred distances (mm) between each recording point E (E_x_, E_y_, E_z_) and the origin of the recording reference system C_3_ (0, 0, 0). We represented ΔV and EF at each of the four recording depths as four perfect concentric spheres (Z_0_, Z_33_, Z_66_, Z_100_), on which we projected the 10–20 points from Z_0_ (where the points were marked). To note, we were not able to register ΔV in the recording points directly under the stimulation electrodes (C_3_ and C_4_ for CONDITON A; Fp_1_, Fp_2_, O_1_, and O_2_ for CONDITON B; F_7_, F_8_, T_5_, and T_6_ for CONDITON C) due to the presence of the stimulating pads; therefore, for these values at Z_0_, Z_33_, Z_66_, and Z_100_, we averaged the surrounding ΔV at each depth. Also, here, we report the numerical and graphical results referring only to CONDITION A, while those from CONDITION B and CONDITION C are reported in the Supplementary Materials.

### 3.1. CONDITION A (Monopolar Montage)

**Figure 4:**
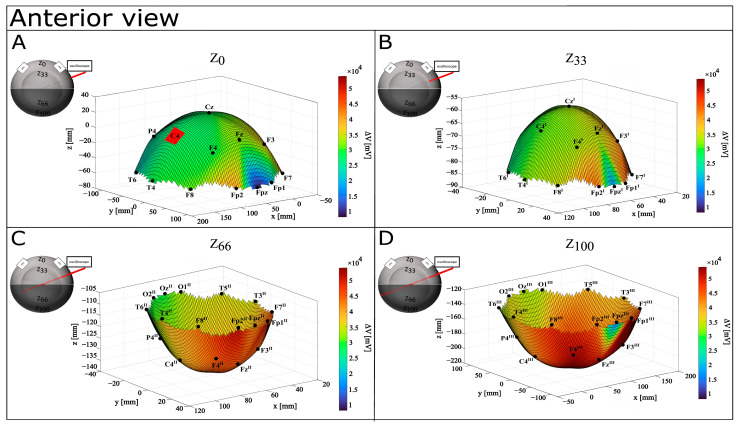
Graphical representation of ΔV distribution in CONDITION A—anterior view. Warm colors refer to a higher value of ΔV recorded, while cool colors refer to a lower value of recorded ΔV. (**A**) Depth Z_0_; (**B**) depth Z_33_; (**C**) depth Z_66_; (**D**) depth Z_100_.

**Figure 5:**
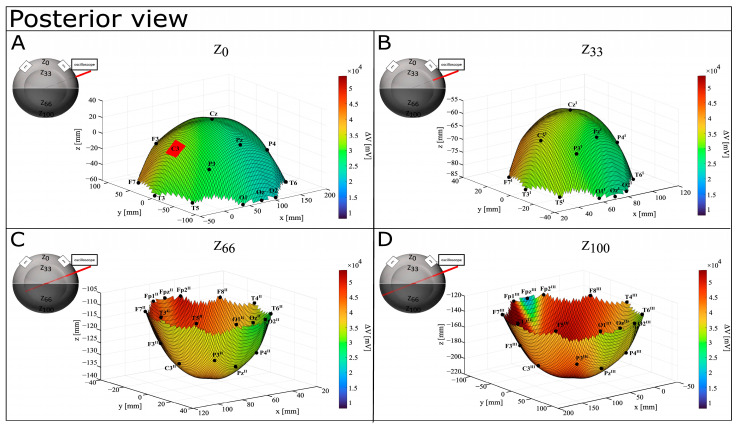
Graphical representation of ΔV distribution in CONDITION A—posterior view. Warm colors refer to a higher value of recorded ΔV recorded, while cool colors refer to lower values of recorded ΔV. (**A**) Depth Z_0_; (**B**) depth Z_33_; (**C**) depth Z_66_; (**D**) depth Z_100_.

**Figure 6:**
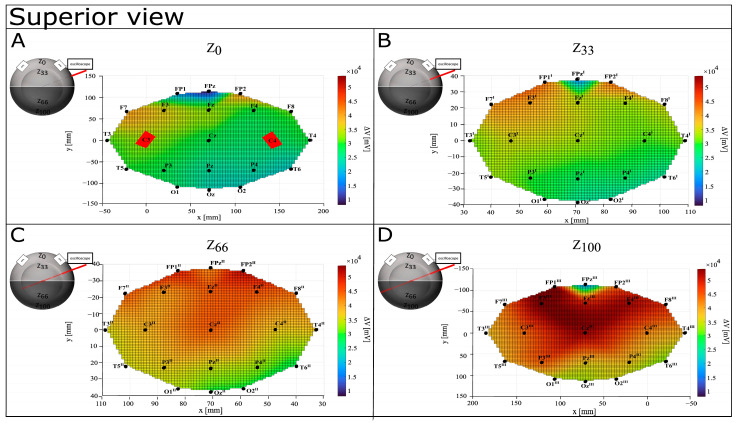
Graphical representation of ΔV distribution in CONDITION A—superior view. Warm colors refer to a higher value of recorded ΔV, while cool colors refer to lower values of recorded ΔV. (**A**) Depth Z_0_; (**B**) depth Z_33_; (**C**) depth Z_66_; (**D**) depth Z_100_.

Table 1 reports values of ΔV (mV) and EF (mV/mm) for each recording point on the perfect spheres. A graphical representation of ΔV distribution shows that the difference in voltage increases with depth, with an anterior-to-posterior shift (see Figure 4, Figure 5 and Figure 6). At the surface, maximal values of ΔV were recorded in Fp2 (39,600 mV) and F7 (39,200 mV), but interestingly, Fp1 (15,800 mV) and Fpz (8080 mV) showed minimal differences in voltage. Although a similar pattern can be identified at Z_33_, deeper levels show a more widespread ΔV distribution. However, at Z_66_, it shifts more posteriorly (with Cz^III^, Fp1^III^, and Fp2^III^ showing higher values, respectively, of 43,600 mV, 48,400 mV, and 50,400 mV), and at Z_100_, it concentrates around F3^III^ (54,000 mV), Cz^III^ (52,000 mV), and F4^III^ (51,600 mV). Notably, Z_100_ presents a pattern of ΔV distribution similar to Z_0_ and Z_33_, with the lowest value recorded in FP2^III^ (16,600 mV).

**Figure 7:**
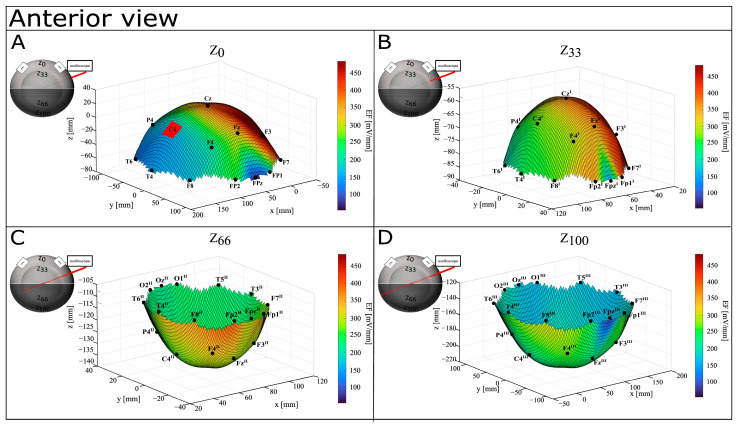
Graphical representation of EF distribution in CONDITION A—anterior view. Warm colors refer to a higher value of calculated EF, while cool colors refer to lower values of calculated ΔV. (**A**) Depth Z_0_; (**B**) depth Z_33_; (**C**) depth Z_66_; (**D**) depth Z_100_.

**Figure 8:**
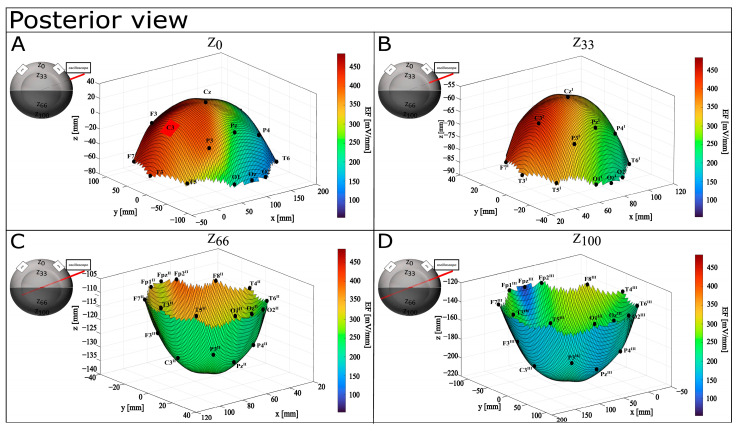
Graphical representation of EF distribution in CONDITION A—posterior view. Warm colors refer to a higher value of calculated EF, while cool colors refer to lower values of calculated ΔV. (**A**) Depth Z_0_; (**B**) depth Z_33_; (**C**) depth Z_66_; (**D**) depth Z_100_.

**Figure 9:**
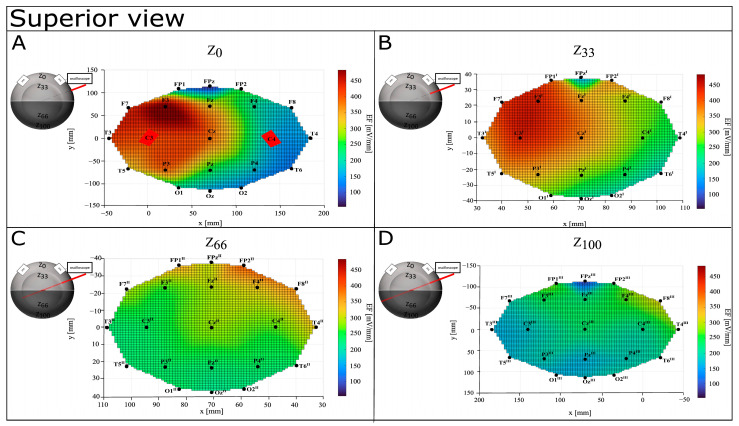
Graphical representation of EF distribution in CONDITION A—superior view. Warm colors refer to a higher value of calculated EF, while cool colors refer to lower values of calculated ΔV. (**A**) Depth Z_0_; (**B**) depth Z_33_; (**C**) depth Z_66_; (**D**) depth Z_100_.

A graphical representation of EF distribution shows a tendency to be focalized in the left hemisphere, also while increasing the depth (see Figure 7, Figure 8 and Figure 9). On the surface, higher values (from 318.59 mV/mm to 484.73 mV/mm) are localized in the left fronto–parietal zone, with lower ones on the anterior (Fp1, Fpz—122.13 mV/mm and 54.79 mV/mm) and posterior (O1, Oz—204.07 mV/mm and 154.62 mV/mm) portions of the hemisphere. Although still mainly present in the anterior part of the left hemisphere (from 330.48 mV/mm to 436.55 mV/mm), the distribution is more spread at Z_33_, and it shifts more posteriorly with the highest values around 354.68 mV/mm at FP2^II^, Z_66_. At Z_100_, the distribution is lightly concentrated in the postero–lateral portion of the left hemisphere (F8^III^), with FP2^III^ showing the lowest values (208.77 mV/mm).

### 3.2. CONDITION B (Bipolar Antero–Posterior Montage)

The Supplementary Materials report numerical and graphical representations of the CONDITION B results. Table S3 reports values of ΔV (mV) and EF (mV/mm) for each recording point on the perfect spheres. A graphical representation of ΔV distribution shows an evident pattern in which the highest values of the difference in voltage are concentrated in the anterior hemisphere. In particular, at Z_0_, maximal values (from 26,900 mV to 33,800 mV) amassed around the F7, F3, Fz, T4, F8, T3, C3, Cz, and C4, while at Z_33_, the distribution, although localized in the anterior hemisphere, is more widespread. At Z_66_ and Z_100_, a latero–lateral pattern of distribution can be identified, with maximal values in T3^II^ (33200 mV) and T3^III^ (33,400 mV) (see Figures S1–S3). A graphical representation of EF distribution shows the tendency to be amassed in the left postero–lateral zones, particularly at Z_0_ and Z_100_ (see Figures S4–S6). Indeed, at the surface, the maximal value was recorded at Oz (435.44 mV/mm) but high values were found also around T5 (325 mV/mm), P3 (318.35 mV/mm), and Pz (266,46 mV/mm). At Z_33_ and Z_66_, distribution is way more widespread, although still localized in the posterior hemisphere, and mainly on the left side. Interestingly, at Z_100_, distribution became extremely focalized at FP2^III^ and FPz^III^, with values of 253.67 mV/mm and 326.93 mV/mm.

### 3.3. CONDITION C (Bipolar Latero–Lateral Montage)

The Supplementary Materials report numerical and graphical representations of the CONDITION C results. Table S4 reports values of ΔV (mV) and EF (mV/mm) for each recording point on the perfect spheres. A graphical representation of ΔV distribution shows an evident pattern in which the highest values of the difference in voltage are concentrated in the left hemisphere at Z_0_ (T3, C3, F3, Fp1—max value 52,000 mV), tend to be more widespread at Z_33_ (but still more present in the left portion of the sphere), and then mass around Fp1^II^ and Fp2^II^ at Z_66_ (48,000 mV and 47,600 mV) and around Fpz^III^ and T4^III^ at Z_100_ (48,800 mV and 47,200 mV) (see Figures S7–S9). A graphical representation of EF distribution shows the tendency to be amassed in the right postero–lateral zones, particularly at Z_0_ and Z_100_ (see Figures S10–S12). Indeed, at the surface, the maximal value was recorded at P4 (643 mV/mm) but high values were found also around T4 (553.91 mV/mm), C4 (426.22 mV/mm), and O_2_ (423.2 mV/mm). At Z_33_ and Z_66_, distribution is far more widespread, although still localized in the posterior hemisphere, mainly on the right side. Interestingly, at Z_100_, distribution became extremely focalized at F7^III^, with values of 227.39 mV/mm.

## 4. Discussion

### 4.1. Validation Experiments

In this study, we aimed to qualitatively shape tDCS-induced EFs in a physical phantom head model (i.e., a watermelon), recording signals under three stimulation conditions that were suggested and developing significant stimulation in deep brain structures. Increasing the depth, we found that monopolar stimulation (CONDITION A) resulted in a focused EF distribution with a tendency to shift in the left-to-right direction; anterior-to-posterior stimulation (CONDITION B) created an EF distribution localized in the left parieto–occipital zones but only at the surface and the maximal depth of recording. Similarly, right-to-left stimulation (CONDITION C) showed an EF distribution amassing around the right parieto–occipital zones but only at the surface and the maximal depth of recording. It is noteworthy that CONDITION B and CONDITION C exhibited an almost perfectly symmetrical pattern of EF distribution. As watermelon might be thought of as a reasonable biophysical approximation of the human head [29], we chose to calculate EF according to the 10–20 system to simulate human brain cortical zones and record at four depths (surface, at 33% of the diameter, at 66% of the diameter, and at 100% of the diameter) to resemble anatomical head structures (respectively, human skin, human cortex, human diencephalon and midbrain, and human brainstem). In this view, our results suggest that for all conditions, EF is massively shunted by the skin. Although in line with previous knowledge [42], these results might reflect the displacement of conductive gel on the surface of the watermelon. In CONDITON A, cortical stimulation was widespread but specifically localized at the left fronto–parietal zones, and the diencephalon and brainstem could be reached by induced EF. For CONDITION B and CONDITION C, the left and right (respectively) temporo–occipital cortex were more stimulated, with surprisingly high EF values at the level of the human brainstem (more on the left for CONDITION B, and more on the right for CONDITION C), therefore suggesting a potential in stimulating the deepest cerebral zones. However, being that this is a first-of-its-kind study, our results might be compared only with the findings from computational models [45,46,47,48,49]. Previous studies have matched computational with physical results [14,21,27] but with the aim to validate in silico findings. Therefore, the authors replicated in the real world the characteristics of the simulation [14,21,27]. One should keep in mind that in this case, a frank comparison needs to be carefully considered due to the different intrinsic (e.g., computational models rely on tissue conductivities arbitrarily assigned; phantom models suffer from technical and methodological limitations of recording) and extrinsic (e.g., different study settings) characteristics of the models. The main pitfall might come from our method of signal acquisition, i.e., having the reference for recording attached directly to the stimulating surface of the tDCS electrode in C_3_ (CONDITION A), O_1_ (CONDITION B), and T_6_ (CONDITION C). The tDCS montage we simulated in CONDITION A (i.e., two anodes over C3 and C4; reference in extracephalic position) was suggested to induce a concentration of currents when compared to cephalic montages [45,46,49]. Although the computational results are still a matter of debate [50], our experiments suggest that CONDITION A developed a clear concentration of EF when compared with cephalic montages (CONDITION B and CONDITION C). This pattern is evident on the surface (human skin), at Z_33_ (human cortex), and at Z_66_ (human diencephalon and midbrain), but not at Z_100_ (human brainstem), where cephalic montages induced an almost punctual EF. Also, it was suggested that the extracephalic reference would result in a substantially greater depth of stimulation compared to cephalic configurations [45,46,47,48], although the matter remains controversial [50,51]. For example, Parazzini et al., 2013 showed that J during tDCS with an active electrode over C3 and C4 and an extracephalic reference was always higher in the midbrain, pons, and medulla than in cephalic montages [48]. These areas would roughly correspond to recordings in Z_66_ and Z_100_ in CONDITION A. Indeed, our experiments suggest that CONDITION A developed a higher E in Z_66_, compared to CONDITION B and CONDITION C. Also, at Z_100_, the monopolar montage induced weaker EF, but it was more diffuse and widespread. On the other hand, there is not much literature at present on the tDCS montages we simulated in CONDITION B (anodes over Fp1 and Fp2; references over O_1_ and O_2_) and CONDITION C (anodes over F7 and T5; references over F8 and T6). However, we applied these stimulation montages to assess the effect of stimulating electrode distance on EF distribution, with four cephalic electrodes. Our results suggest that these montages, compared to monopolar, developed lower values of EF at Z_0_, Z_33_, and Z_66_, but with higher and clearly localized EF at Z_100_. This suggests that, although globally inferior, CONDITION B and CONDITION C montages might induce a deep and focalized stimulation but only at the deepest brain levels.

### 4.2. General Considerations and Limitations

Determining the EF induced in the brain by tDCS is of particular concern, for both ethical and clinical implications. As watermelon might be thought of as a reasonable biophysical approximation of the human head [29], in this first-of-its-kind study, we suggest that it can be used as a cheap, feasible, and easy phantom head model to characterize the EFs induced by tDCS and, potentially, even other non-invasive brain stimulation techniques. Also, we described a methodological protocol to standardize the experiments, ensure homogenous signal recording and processing, and facilitate the translation of the results. However, several limitations must be considered. First, phantom models such as watermelons are similar but not identical to the human head, and caution is required when interpreting phantom findings. Not only EFs but also the head anatomy determine EF distribution and effects, as disclosed by a number of computational models [52,53,54,55]. For example, skull thickness and composition determine the amount of current reaching the brain [42,56]; cortical folding affects the polarity of the stimulation, creating a highly mixed pattern of directionality [57]; and cerebrospinal fluid, which dissipates the current to the deep regions [42]. Watermelon has a simpler anatomy and cannot account for human complexity. First, phantom models such as watermelons are similar, but not identical to human head and caution is required when interpreting phantom findings. Not only EFs, but also the head anatomy determines EFs distribution and effects, as disclosed by a number of computational models [52,53,54,55]. For example, skull thickness and composition determines the amount of current reaching the brain [42,56], cortical foldings affect the polarity of the stimulation creating a highly mixed pattern of directionality [57], and cerebrospinal fluid dissipates the current to the deep regions [42]. Watermelon has a far simpler anatomy, and in phantom recordings cannot account for human complexity. Second, recording errors might have occurred, as imprecisions in the measurement of potentials are unavoidable and greatly influence the estimation procedure [6,14]. For example, discrepancies in the electrode–tissue interface due to the presence of air or electrode inclination [6] or unavoidably small displacements of measurement points [28] are hurdles intrinsic to the methodology [6]. Also, the presence of measurement electrodes themselves, holes, saline solution, and/or conductive gel on the surface of the watermelon might have modified the electric conductivity and/or EF distribution, as occurs for other models and in vivo recordings [26]. Third, we calculated the value of EF according to the formula EF = ∆V/ΔS, which is an approximate average value with respect to integral calculations (EF = dV/dS). We opted for this solution first because our aim was to provide a simple and practical methodology that could be adopted also by non-expert clinicians in electrical field calculations, and, second, because we assumed that the watermelon could be considered an isotropic and homogeneous means. Finally, the decision to graphically represent the results on a perfect sphere could have slightly distorted the spatial location of the recording points in the process of approximation from the real shape of each watermelon.

## 5. Conclusions

Determining the EF induced in the brain by tDCS is of particular concern, for both ethical and clinical implications. In this first-of-its-kind study, we characterized tDCS-induced Es in a phantom model (i.e., a watermelon). We found that despite the physical approximations to a human head and the methodological limitations of this study, watermelon might be considered a reliable human head phantom model for the prediction of tDCS-induced EFs, which is of key importance for the study of neuromodulation. Further studies are needed to better understand whether watermelon might be considered an affordable but reliable phantom head model that is potentially useful to describe in depth the characterization of tDCS-induced EFs, now that recent advancements in technology (e.g., triboelectric nanogenerators, which transform mechanical movement into electrical energy [58]) promise to increase the feasibility and user-friendliness of neurostimulation techniques.

## Figures and Tables

**Table 1:** Translation of each recording electrode E (E_x_, E_y_, E_z_) on a sphere with origin C3 (C3x, C3y, C3z). Distance between each recording point E (E_x_, E_y_, E_z_) at each depth Z and C3 (C3x, C3y, C3z), with recorded ΔV and inferred EF reported.

Electrode Placement on Surface	Depth Z	X (mm)	Y (mm)	Z (mm)	C3E	ΔV (mV)	EF (mV/mm)
Fp_1_	Z_0_	35.38	108.90	−60.20	129.37	15,800.00	122.13
	Z_33_	58.97	36.30	−85.00	109.64	41,200.00	375.77
	Z_66_	58.97	−36.30	−109.81	129.82	48,400.00	372.82
	Z_100_	35.38	−108.90	−134.61	176.73	53,200.00	301.03
Fp_z_	Z_0_	70.77	114.51	−60.20	147.46	8080.00	54.79
	Z_33_	70.77	38.17	−85.00	117.01	15,600.00	133.32
	Z_66_	70.77	−38.17	−109.81	136.10	41,600.00	305.66
	Z_100_	70.77	−114.51	−134.61	190.37	16,600.00	87.20
Fp_2_	Z_0_	106.15	108.90	−60.20	163.56	39,600.00	242.11
	Z_33_	82.56	36.30	−85.00	123.94	42,800.00	345.34
	Z_66_	82.56	−36.30	−109.81	142.10	50,400.00	354.68
	Z_100_	106.15	−108.90	−134.61	203.10	42,400.00	208.77
F_7_	Z_0_	−21.87	67.31	−60.20	92.91	39,200.00	421.91
	Z_33_	39.89	22.44	−85.00	96.54	40,800.00	422.62
	Z_66_	39.89	−22.44	−109.81	118.96	44,000.00	369.86
	Z_100_	−21.87	−67.31	−134.61	152.08	42,400.00	278.80
F_3_	Z_0_	13.52	78.80	−26.64	84.27	35,600.00	484.73
	Z_33_	51.68	26.27	−73.82	93.86	39,600.00	436.23
	Z_66_	51.68	−26.27	−121.00	134.17	40,400.00	291.43
	Z_100_	13.52	−78.80	−168.17	186.21	54,000.00	275.85
F_7_	Z_0_	70.77	70.77	0.00	100.08	32,000.00	319.73
	Z_33_	70.77	23.59	−64.94	98.90	34,400.00	347.82
	Z_66_	70.77	−23.59	−129.87	149.77	44,000.00	293.78
	Z_100_	70.77	−70.77	−194.81	219.02	49,600.00	226.47
F_4_	Z_0_	128.02	78.80	−26.64	152.67	29,200.00	208.24
	Z_33_	89.85	26.27	−73.82	119.22	33,600.00	294.73
	Z_66_	89.85	−26.27	−121.00	152.98	44,400.00	286.76
	Z_100_	128.02	−78.80	−168.17	225.57	51,600.00	225.01
F_8_	Z_0_	163.41	67.31	−60.20	186.70	31,600.00	169.26
	Z_33_	101.65	22.44	−85.00	134.39	35,600.00	264.90
	Z_66_	101.65	−22.44	−109.81	151.31	42,800.00	282.87
	Z_100_	163.41	−67.31	−134.61	222.15	47,600.00	214.27
T_3_	Z_0_	−43.74	0.00	−60.20	74.41	30,800.00	413.92
	Z_33_	32.60	0.00	−85.00	91.04	32,000.00	351.49
	Z_66_	32.60	−0.00	−109.81	114.54	35,200.00	307.30
	Z_100_	−43.74	−0.00	−134.61	141.54	36,800.00	260.00
C_3_	Z_0_	−0.00	0.00	0.00	0.00	N.R. *	404.08
	Z_33_	47.18	0.00	−64.94	80.27	N.R. *	436.54
	Z_66_	47.18	−0.00	−129.87	138.18	N.R. *	275.59
	Z_100_	−0.00	−0.00	−194.81	194.81	N.R. *	219.70
C_z_	Z_0_	70.77	0.00	22.99	74.41	28,000.00	376.29
	Z_33_	70.77	0.00	−57.27	91.04	33,200.00	364.67
	Z_66_	70.77	0.00	−137.54	154.68	43,600.00	281.88
	Z_100_	70.77	0.00	−217.81	229.01	52,000.00	227.06
C_4_	Z_0_	141.54	0.00	0.00	141.54	N.R. *	0.00
	Z_33_	94.36	0.00	−64.94	114.54	N.R. *	267.49
	Z_66_	94.36	0.00	−129.87	160.53	N.R. *	229.73
	Z_100_	141.54	0.00	−194.81	240.80	N.R. *	170.76
T_4_	Z_0_	185.28	0.00	−60.20	194.81	29,200.00	149.89
	Z_33_	108.94	0.00	−85.00	138.18	32,000.00	231.59
	Z_66_	108.94	0.00	−109.81	154.68	36,800.00	237.91
	Z_100_	185.28	0.00	−134.61	229.01	38,000.00	165.93
T_5_	Z_0_	−21.87	−67.31	−60.20	92.91	29,600.00	318.59
	Z_33_	39.89	−22.44	−85.00	96.54	32,800.00	339.75
	Z_66_	39.89	22.44	−109.81	118.96	34,800.00	292.53
	Z_100_	−21.87	67.31	−134.61	152.08	37,600.00	247.24
P_3_	Z_0_	13.52	−78.80	−26.64	84.27	28,000.00	381.25
	Z_33_	51.68	−26.27	−73.82	93.86	30,000.00	330.48
	Z_66_	51.68	26.27	−121.00	134.17	36,000.00	259.69
	Z_100_	13.52	78.80	−168.17	186.21	43,200.00	220.68
P_z_	Z_0_	70.77	−70.77	0.00	100.08	24,400.00	243.80
	Z_33_	70.77	−23.59	−64.94	98.90	27,600.00	279.06
	Z_66_	70.77	23.59	−129.87	149.77	34,400.00	229.68
	Z_100_	70.77	70.77	−194.81	219.02	36,700.00	167.57
P_4_	Z_0_	128.02	−78.80	−26.64	152.67	24,000.00	171.16
	Z_33_	89.85	−26.27	−73.82	119.22	27,200.00	238.59
	Z_66_	89.85	26.27	−121.00	152.98	32,400.00	209.26
	Z_100_	128.02	78.80	−168.17	225.57	36,800.00	160.47
T_6_	Z_0_	163.41	−67.31	−60.20	186.70	21,600.00	115.69
	Z_33_	101.65	−22.44	−85.00	134.39	24,800.00	184.53
	Z_66_	101.65	22.44	−109.81	151.31	28,000.00	185.06
	Z_100_	163.41	67.31	−134.61	222.15	31,600.00	142.24
O_1_	Z_0_	35.38	−108.90	−60.20	129.37	26,400.00	204.07
	Z_33_	58.97	−36.30	−85.00	109.64	28,800.00	262.67
	Z_66_	58.97	36.30	−109.81	129.82	33,200.00	255.74
	Z_100_	35.38	108.90	−134.61	176.73	33,200.00	187.86
O_z_	Z_0_	70.77	−114.51	−60.20	147.46	22,800.00	154.62
	Z_33_	70.77	−38.17	−85.00	117.01	25,200.00	215.37
	Z_66_	70.77	38.17	−109.81	136.10	29,200.00	214.55
	Z_100_	70.77	114.51	−134.61	190.37	33,200.00	174.40
O_2_	Z_0_	106.15	−108.90	−60.20	163.56	22,000.00	134.51
	Z_33_	82.56	−36.30	−85.00	123.94	24,400.00	196.88
	Z_66_	82.56	36.30	−109.81	142.10	28,800.00	202.68
	Z_100_	106.15	108.90	−134.61	203.10	34,800.00	171.35

N.R. = not recordable; * values of ΔV were calculated as average of surrounding ΔVs at each depth.

## Data Availability

The original contributions presented in this study are included in the article/Supplementary Materials. Further inquiries can be directed to the corresponding author(s).

## Supplementary Materials

The following supporting information can be downloaded at: https://www.mdpi.com/article/10.3390/bioengineering12040346/s1, Practical application (Watermelon preparation and tDCS protocols; Recording acquisition; Graphical representation; Results for CONDITION B and CONDITION C; Table S1: Physical characteristics of phantom models; Table S2: Polar (ρ, φ, θ) and cartesian (x, y, z) coordinates for recording electrodes E at each depth; Table S3: Translation of each recording electrodes E (E_x_, E_y_, E_z_) on a sphere with origin O_1_ (O_1x_, O_1y_, O_1z_). Distance between each recording point E (E_x_, E_y_, E_z_) at each depth Z and O_1_ (O_1x_, O_1y_, O_1z_), with ∆V recorded and EF inferred are reported; Table S4: Translation of each recording electrodes E (E_x_, E_y_, E_z_) on a sphere with origin T_6_ (T_6x_, T_6y_, T_6z_). Distance between each recording point E (E_x_, E_y_, E_z_) at each depth Z and T_6_ (T_6x_, T_6y_, T_6z_), with ∆V recorded and EF inferred are reported. Figure S1: Graphical representation of ΔV distribution in CONDITION B—anterior view. (A) refers to depth Z_0_; (B) refers to depth Z_33_; (C) refers to depth Z_66_; (D) refers to depth Z_100_. Figure S2: Graphical representation of ΔV distribution in CONDITION B—posterior view. (A) refers to depth Z_0_; (B) refers to depth Z_33_; (C) refers to depth Z_66_; (D) refers to depth Z_100_. Figure S3: Graphical representation of ΔV distribution in CONDITION B—superior view. (A) refers to depth Z_0_; (B) refers to depth Z_33_; (C) refers to depth Z_66_; (D) refers to depth Z_100_. Figure S4: Graphical representation of EF distribution in CONDITION B—anterior view. (A) refers to depth Z_0_; (B) refers to depth Z_33_; (C) refers to depth Z_66_; (D) refers to depth Z_100_. Figure S5: Graphical representation of EF distribution in CONDITION B—posterior view. (A) refers to depth Z_0_; (B) refers to depth Z_33_; (C) refers to depth Z_66_; (D) refers to depth Z_100_. Figure S6: Graphical representation of EF distribution in CONDITION B—superior view. (A) refers to depth Z_0_; (B) refers to depth Z_33_; (C) refers to depth Z_66_; (D) refers to depth Z_100_. Figure S7: Graphical representation of ΔV distribution in CONDITION C—anterior view. (A) refers to depth Z_0_; (B) refers to depth Z_33_; (C) refers to depth Z_66_; (D) refers to depth Z_100_. Figure S8: Graphical representation of ΔV distribution in CONDITION C—posterior view. (A) refers to depth Z_0_; (B) refers to depth Z_33_; (C) refers to depth Z_66_; (D) refers to depth Z_100_. Figure S9: Graphical representation of ΔV distribution in CONDITION C—superior view. (A) refers to depth Z_0_; (B) refers to depth Z_33_; (C) refers to depth Z_66_; (D) refers to depth Z_100_. Figure S10: Graphical representation of EF distribution in CONDITION C—anterior view. (A) refers to depth Z_0_; (B) refers to depth Z_33_; (C) refers to depth Z_66_; (D) refers to depth Z_100_. Figure S11: Graphical representation of EF distribution in CONDITION C—posterior view. (A) refers to depth Z_0_; (B) refers to depth Z_33_; (C) refers to depth Z_66_; (D) refers to depth Z_100_. Figure S12: Graphical representation of EF distribution in CONDITION C—superior view. (A) refers to depth Z_0_; (B) refers to depth Z_33_; (C) refers to depth Z_66_; (D) refers to depth Z_100_.
