# Supplementary material for: The “Cocombola Study”: A Physical Phantom Model for tDCS-Induced Electric Field Distribution
Source: Bioengineering (Basel). 2025 Mar 27;12(4):346. doi: 10.3390/bioengineering12040346 (PMC12024709; doi:10.3390/bioengineering12040346)
Supplement: Supplementary file 1 [file bioengineering-12-00346-s001.zip › bioengineering-3456709-supplementary.pdf]

Practical application

For the practical examples we report, we used a watermelon (Citrullus lanatus). For each stimulation condition (CONDITION A, CONDITION B, and CONDITION C), we considered a watermelon (n=3) whose shape and weight were similar to average human head (i.e., around 4 kg [1]) (see Table S1 for details).

Watermelon preparation and tDCS protocols

After creating the cranial holes, marking the 10-20 system electrodes positions on the peel and placing the watermelon into the plastic container with water, we applied three different tDCS montages. In the first condition (CONDITION A), we simulated a monopolar montage – two anodes over motor cortices, reference over right deltoid. Therefore, we placed two anodes (7 × 5 cm^2) over C_3 and C_4 (corresponding respectively to human left and right motor cortex) and the reference (8 x 6 cm^2) in the water (resembling the extracephalic position). For CONDITION B, two anodes (5 × 5 cm^2) were placed over Fp_1 and Fp_2 (corresponding respectively to human left and right prefrontal cortex) and two reference (5 × 5 cm^2) over O_1 and O_2 (corresponding respectively to human left and right occipital cortex). For CONDITION C, two anodes (5 × 5 cm^2) were placed over F_7 and T_5 (corresponding respectively to human left ventrolateral prefrontal and occipitotemporal cortex) and references (5 × 5 cm^2) over F_8 and T_6 (corresponding respectively to human right ventrolateral prefrontal and occipitotemporal cortex). DC stimulation was applied using a stimulator (CONDITION A) or two stimulators (CONDITION B and C) (HDCStim, Newronika, Italy) through silicone rubber pad electrodes (1mm thickness), with conductive gel applied between the electrodes and the peel to lower tDCS electrode resistance. Since the purpose of the study was to record tDCS-induced ΔV and calculate EF, DC was delivered with a ramp-up and ramp-down of just 5 s to allow recordings when the intensity was steady. In order to keep the current density (current strength divided by electrode size) constant in all the conditions (current density = 0.028 mA/cm^2) [2], we set the intensity of stimulation at 2mA in CONDITION A, and 1.4 mA in CONDITION B and CONDITION C.

Recording acquisition

The signal was recorded from 2 self-adhesive bipolar surface Ag/AgCl electrodes (15 x 20 mm) connected to a digital oscilloscope (Tektronix TDS2024C oscilloscope - Tektronix, Inc., Johnston, IA, USA). One recording electrode was attached directly to the stimulating surface of tDCS electrode in C_3 (CONDITION A), O_1 (CONDITION B) and T_6 (CONDITION C), these being considered references for recording. The other electrode was wrapped around one end of a 48.5 cm

long and 5 mm thick copper wire, accurately covered with insulating tape. The other end of the copper wire was used as recording area (98.12 mm^2). During stimulation, we recorded ΔV as variation of voltage between the two recording electrodes. Data were saved and stored for off-line analysis. No amplification strategies, band-pass filters or ameliorative acquisition procedures were applied. Measurements and recordings were performed for each CONDITION, following the same steps. Firstly, we assessed the characteristics of each watermelon and marked the electrode placements E according to 10-20 system. Then, ΔV was acquired in all the 21 scalp electrodes, at four depths: on the surface (Z_0), at 33% of the diameter (Z_{33}), at 66% of the diameter (Z_{66}), and at 100% of the diameter (Z_{100}). After recording, the holes were filled with saline solution. In all conditions, signal acquisition occurred when DC stimulation intensity was maximal and steady.

Graphical representation

To represent the results in a simple and comparable graph, we approximated the watermelon in CONDITON A, CONDITION B, and CONDITION C to perfect spheres with the same radius and electrodes' relative position, as previously reported [3].

We calculated the 3D coordinates of each recording point E (E_x, E_y, E_z) at Z_0, Z_{33}, Z_{66}, and Z_{100} by using polar coordinates (ρ, φ, θ) according to the formula:

$$\rho = | R - Z \cdot D | \qquad (1)$$

where R is the radius and D is the diameter of the watermelon, and Z set at 0, 0.33, 0.66 and 1 for each depth, respectively. For each recording point, polar coordinates ϕ (i.e., the angles between the r vector and the x axis on the (x, y) plane) and θ (i.e., the angles between the ρ vector and the z axis on the (y, z) plane) are calculated by applying trigonometry to the 10-20 conventional system [43] built on a perfect sphere. The system, indeed, considers specific ratios in measurements of surface recording points [43], and allow to divide the sphere in known angles. Then, we switched to the cartesian coordinates (x, y, z) of the recording points E in the sphere centered in the origin O (O_x = 0; O_y = 0; O_z = 0) by considering the relations:

$$\begin{cases} x = \rho sin(\phi) cos(\theta) \\ y = \rho sin(\phi) sin(\theta) \\ z = \rho cos(\phi) \end{cases} \qquad (2)$$

Where $\rho \in [0, +\infty)$, $\theta \in [0, 2\pi)$, $\varphi \in [0, 2\pi]$.

Table S2 summarizes the polar (ρ, ϕ, θ) and cartesian (x, y, z) coordinates computed for each electrode E at each depth Then, we translated the obtained sphere model into the sphere model centered in the references for the recording system R (Rx, Ry, Rz), as subtraction of cartesian

coordinates of R (R_x, R_y, R_z) to the respective cartesian coordinates of each recording point E (E_x, E_y, E_z). Finally, to estimate EF in each recording point E (E_x, E_y, E_z), we calculated the distance between each recording point E (E_x, E_y, E_z) at each depth Z and the references for the recording system R (R_x, R_y, R_z), i.e. the distance $\overline{RE}$ (see figure S1 – C), according to the equations:

$$\overline{RE} = \sqrt{{E_x}^2 + {E_y}^2 + {E_z}^2}$$

EF was calculated as ratio between the ΔV (mV) experimentally recorded and the inferred distances (mm) between each recording point E (E_x, E_y, E_z) and R (0, 0, 0). We represented ΔV and EF at each of the four recording depths as four perfect concentric spheres (Z_0, Z_{33}, Z_{66}, Z_{100}), on which we projected the 10-20 points from Z_0 where points were marked. To note, we could not register ΔV in the recording points under the stimulating pads; therefore, for these values at Z_0, Z_{33}, Z_{66}, and Z_{100}, we average the surrounding ΔV at each depth. The reference values for each CONDITION are: C_3 (C_{3x}, C_{3y}, C_{3z}) for CONDITON A, O1 (O_{1x}, O_{1y}, O_{1z}) for CONDITION B, and T_6 (T_{6x}, T_{6y}, T_{6z}). The distance RE was therefore: $\overline{C_3E}$ for the CONDITION A, the distance $\overline{O_1E}$ for the CONDITION B and the distance $\overline{T_6E}$ for the CONDITION C.

Results for CONDITION B and CONDITION C

Table S3 reports values of ΔV (mV) and EF (mV/mm) for each recording point on the perfect spheres in CONDITION B. Graphical representation of ΔV distribution is reported Figure S1, S2, S3. Graphical representation of EF distribution is reported Figure S4, S5, S6.

Table S4 reports values of ΔV (mV) and EF (mV/mm) for each recording point on the perfect spheres in CONDITION C. Graphical representation of ΔV distribution is reported Figure S7, S8, S9. Graphical representation of EF distribution is reported Figure S10, S11, S12.

Table S1. Physical characteristics of phantom models

PHANTOM MODEL	*Weight (Kg)*	*Max diameter (mm)*	*Temperature (peel) (°C)*	*Temperature (pulp) (°C)*	*Resistence (peel) (kΩ)*	*Resisence (pulp) (kΩ)*
Watermelon CONDITIO N A	4.83	22	22.7	18.5	200	180
Watermelon CONDITIO N B	5.12	22	22.8	22.3	530	250
Watermelon CONDITIO N C	5.03	21	23.5	23.2	260	150

Table S2. Polar (ρ, ϕ, θ) and cartesian (x, y, z)coordinates for recording electrodes E at each depth

Electrode placement according to 10-20 system	*depth*	*r (mm)*	*θ (rad)*	*ϕ (rad)*	*x (mm)*	*y (mm)*	*z (mm)*
Fp_1	Z_0	120,40	1,885	1,257	-35,38	108,90	37,21
	Z_{33}	40,133	1,885	1,257	-11,79	36,30	12,40
	Z_{66}	40,133	-1,885	1,885	-11,79	-36,30	-12,40
	Z_{100}	120,40	-1,885	1,885	-35,38	-108,90	-37,21
Fp_z	Z_0	120,40	1,571	1,257	-0,00	114,51	37,21
	Z_{33}	40,133	1,571	1,257	-0,00	38,17	12,40
	Z_{66}	40,133	-1,571	1,885	-0,00	-38,17	-12,40
	Z_{100}	120,40	-1,571	1,885	-0,00	-114,51	-37,21
Fp_2	Z_0	120,40	1,257	1,257	35,38	108,90	37,21
	Z_{33}	40,133	1,257	1,257	11,79	36,30	12,40
	Z_{66}	40,133	-1,257	1,885	11,79	-36,30	-12,40
	Z_{100}	120,40	-1,257	1,885	35,38	-108,90	-37,21
F_7	Z_0	120,40	2,513	1,257	-92,64	67,31	37,21
	Z_{33}	40,133	2,513	1,257	-30,88	22,44	12,40
	Z_{66}	40,133	-2,513	1,885	-30,88	-22,44	-12,40

	Z_{100}	120,40	-2,513	1,885	-92,64	-67,31	-37,21
F_3	Z_0	120,40	2,199	0,942	-57,25	78,80	70,77
	Z_{33}	40,133	2,199	0,942	-19,08	26,27	23,59
	Z_{66}	40,133	-2,199	2,199	-19,08	-26,27	-23,59
	Z_{100}	120,40	-2,199	2,199	-57,25	-78,80	-70,77
F_z	Z_0	120,40	1,571	0,628	-0,00	70,77	97,41
	Z_{33}	40,133	1,571	0,628	-0,00	23,59	32,47
	Z_{66}	40,133	-1,571	2,513	-0,00	-23,59	-32,47
	Z_{100}	120,40	-1,571	2,513	-0,00	-70,77	-97,41
F_4	Z_0	120,40	0,942	0,942	57,25	78,80	70,77
	Z_{33}	40,133	0,942	0,942	19,08	26,27	23,59
	Z_{66}	40,133	-0,942	2,199	19,08	-26,27	-23,59
	Z_{100}	120,40	-0,942	2,199	57,25	-78,80	-70,77
F_8	Z_0	120,40	0,628	1,257	92,64	67,31	37,21
	Z_{33}	40,133	0,628	1,257	30,88	22,44	12,40
	Z_{66}	40,133	-0,628	1,885	30,88	-22,44	-12,40
	Z_{100}	120,40	-0,628	1,885	92,64	-67,31	-37,21
T_3	Z_0	120,40	3,142	1,257	-114,51	0,00	37,21
	Z_{33}	40,133	3,142	1,257	-38,17	0,00	12,40
	Z_{66}	40,133	-3,142	1,885	-38,17	-0,00	-12,40
	Z_{100}	120,40	-3,142	1,885	-114,51	-0,00	-37,21
C_3	Z_0	120,40	3,142	0,628	-70,77	0,00	97,41
	Z_{33}	40,133	3,142	0,628	-23,59	0,00	32,47
	Z_{66}	40,133	-3,142	2,513	-23,59	-0,00	-32,47
	Z_{100}	120,40	-3,142	2,513	-70,77	-0,00	-97,41
C_z	Z_0	120,40	0,000	0,000	0,00	0,00	120,40
	Z_{33}	40,133	0,000	0,000	0,00	0,00	40,13
	Z_{66}	40,133	0,000	3,142	0,00	0,00	-40,13
	Z_{100}	120,40	0,000	3,142	0,00	0,00	-120,40
C_4	Z_0	120,40	0,000	0,628	70,77	0,00	97,41
	Z_{33}	40,133	0,000	0,628	23,59	0,00	32,47
	Z_{66}	40,133	0,000	2,513	23,59	0,00	-32,47

	Z_{100}	120,40	0,000	2,513	70,77	0,00	-97,41
T_4	Z_0	120,40	0,000	1,257	114,51	0,00	37,21
	Z_{33}	40,133	0,000	1,257	38,17	0,00	12,40
	Z_{66}	40,133	0,000	1,885	38,17	0,00	-12,40
	Z_{100}	120,40	0,000	1,885	114,51	0,00	-37,21
T_5	Z_0	120,40	3,770	1,257	-92,64	-67,31	37,21
	Z_{33}	40,133	3,770	1,257	-30,88	-22,44	12,40
	Z_{66}	40,133	-3,770	1,885	-30,88	22,44	-12,40
	Z_{100}	120,40	-3,770	1,885	-92,64	67,31	-37,21
P_3	Z_0	120,40	4,084	0,942	-57,25	-78,80	70,77
	Z_{33}	40,133	4,084	0,942	-19,08	-26,27	23,59
	Z_{66}	40,133	-4,084	2,199	-19,08	26,27	-23,59
	Z_{100}	120,40	-4,084	2,199	-57,25	78,80	-70,77
P_z	Z_0	120,40	4,712	0,628	-0,00	-70,77	97,41
	Z_{33}	40,133	4,712	0,628	-0,00	-23,59	32,47
	Z_{66}	40,133	-4,712	2,513	-0,00	23,59	-32,47
	Z_{100}	120,40	-4,712	2,513	-0,00	70,77	-97,41
P_4	Z_0	120,40	5,341	0,942	57,25	-78,80	70,77
	Z_{33}	40,133	5,341	0,942	19,08	-26,27	23,59
	Z_{66}	40,133	-5,341	2,199	19,08	26,27	-23,59
	Z_{100}	120,40	-5,341	2,199	57,25	78,80	-70,77
T_6	Z_0	120,40	5,655	1,257	92,64	-67,31	37,21
	Z_{33}	40,133	5,655	1,257	30,88	-22,44	12,40
	Z_{66}	40,133	-5,655	1,885	30,88	22,44	-12,40
	Z_{100}	120,40	-5,655	1,885	92,64	67,31	-37,21
O_1	Z_0	120,40	4,398	1,257	-35,38	-108,90	37,21
	Z_{33}	40,133	4,398	1,257	-11,79	-36,30	12,40
	Z_{66}	40,133	-4,398	1,885	-11,79	36,30	-12,40
	Z_{100}	120,40	-4,398	1,885	-35,38	108,90	-37,21
O_z	Z_0	120,40	4,712	1,257	-0,00	-114,51	37,21
	Z_{33}	40,133	4,712	1,257	-0,00	-38,17	12,40
	Z_{66}	40,133	-4,712	1,885	-0,00	38,17	-12,40

	Z100	120,40	-4,712	1,885	-0,00	114,51	-37,21
O_2	Z0	120,40	5,027	1,257	35,38	-108,90	37,21
	Z33	40,133	5,027	1,257	11,79	-36,30	12,40
	Z66	40,133	-5,027	1,885	11,79	36,30	-12,40
	Z100	120,40	-5,027	1,885	35,38	108,90	-37,21

Table S3. Translation of each recording electrodes $E\ (E_x, E_y, E_z)$ on a sphere with origin $O_1(O_{1x}, O_{1y}, O_{1z})$. Distance between each recording point $E\ (E_x, E_y, E_z)$ at each depth Z and $O_1(O_{1x}, O_{1y}, O_{1z})$, with ΔV recorded and EF inferred are reported.

Electrode placement on surface	depth Z	*x (mm)*	*y (mm)*	*z (mm)*	$\overline{O_1E}$	$\Delta V\ (mV)$	EF (mV/mm)
Fp_1	Z0	-0,00	217,81	0,00	217,81	N.R.*	157,94
	Z33	23,59	145,20	-24,80	149,18	N.R.*	205,45
	Z66	23,59	72,60	-49,61	91,04	N.R.*	287,78
	Z100	-0,00	-0,00	-74,41	74,41	N.R.*	352,77
Fp_z	Z0	35,38	223,41	0,00	226,19	15200,00	67,20
	Z33	35,38	147,07	-24,80	153,29	32400,00	211,37
	Z66	35,38	70,73	-49,61	93,36	27800,00	297,77
	Z100	35,38	-5,60	-74,41	82,59	27000,00	326,93
Fp_2	Z0	70,77	217,81	0,00	229,01	N.R.*	150,21
	Z33	47,18	145,20	-24,80	154,68	N.R.*	195,24
	Z66	47,18	72,60	-49,61	99,79	N.R.*	264,06
	Z100	70,77	-0,00	-74,41	102,69	N.R.*	253,67
F_7	Z0	-57,25	176,21	0,00	185,28	32800,00	177,03
	Z33	4,51	131,34	-24,80	133,74	30000,00	224,32
	Z66	4,51	86,47	-49,61	99,79	24600,00	246,52
	Z100	-57,25	41,60	-74,41	102,69	25800,00	251,24
F_3	Z0	-21,87	187,71	33,56	191,93	33800,00	182,64
	Z33	16,30	135,17	-13,62	136,83	30200,00	225,99
	Z66	16,30	82,64	-60,80	103,88	26000,00	237,57
	Z100	-21,87	30,10	-107,97	114,21	26200,00	203,33
	Z0	35,38	179,67	60,20	192,76	32800,00	170,16

F_z	Z_{33}	35,38	132,49	-4,74	137,22	30000,00	218,63
	Z_{66}	35,38	85,31	-69,67	115,69	26400,00	228,19
	Z_{100}	35,38	38,13	-134,61	144,31	26000,00	180,16
F_4	Z_0	92,64	187,71	33,56	211,99	34400,00	169,11
	Z_{33}	54,47	135,17	-13,62	146,37	29200,00	205,27
	Z_{66}	54,47	82,64	-60,80	116,15	26000,00	217,00
	Z_{100}	92,64	30,10	-107,97	145,42	26400,00	171,35
F_8	Z_0	128,02	176,21	0,00	217,81	32000,00	146,92
	Z_{33}	66,26	131,34	-24,80	149,18	29200,00	195,73
	Z_{66}	66,26	86,47	-49,61	119,70	25200,00	210,52
	Z_{100}	128,02	41,60	-74,41	153,81	24800,00	161,24
T_3	Z_0	-79,12	108,90	0,00	134,61	33200,00	246,64
	Z_{33}	-2,78	108,90	-24,80	111,73	32800,00	293,57
	Z_{66}	-2,78	108,90	-49,61	119,70	33200,00	277,36
	Z_{100}	-79,12	108,90	-74,41	153,81	33400,00	217,15
C_3	Z_0	-35,38	108,90	60,20	129,37	30600,00	236,54
	Z_{33}	11,80	108,90	-4,74	109,64	30000,00	273,62
	Z_{66}	11,80	108,90	-69,67	129,82	29400,00	226,47
	Z_{100}	-35,38	108,90	-134,61	176,73	29800,00	168,62
C_z	Z_0	35,38	108,90	83,19	141,54	29000,00	204,89
	Z_{33}	35,38	108,90	2,93	114,54	29000,00	253,18
	Z_{66}	35,38	108,90	-77,34	138,18	28400,00	205,53
	Z_{100}	35,38	108,90	-157,61	194,81	28000,00	143,73
C_4	Z_0	106,15	108,90	60,20	163,56	26900,00	164,46
	Z_{33}	58,97	108,90	-4,74	123,94	29200,00	235,60
	Z_{66}	58,97	108,90	-69,67	142,10	27800,00	195,64
	Z_{100}	106,15	108,90	-134,61	203,10	27600,00	135,90
T_4	Z_0	149,89	108,90	0,00	185,28	29600,00	159,76
	Z_{33}	73,55	108,90	-24,80	133,74	28200,00	210,86
	Z_{66}	73,55	108,90	-49,61	140,47	27600,00	196,49
	Z_{100}	149,89	108,90	-74,41	199,66	27200,00	136,23
	Z_0	-57,25	41,60	0,00	70,77	23000,00	325,00

T_5	Z_{33}	4,51	86,47	-24,80	90,07	24600,00	273,13
	Z_{66}	4,51	131,34	-49,61	140,47	27000,00	192,22
	Z_{100}	-57,25	176,21	-74,41	199,66	27200,00	136,23
P_3	Z_0	-21,87	30,10	33,56	50,11	20200,00	318,35
	Z_{33}	16,30	82,64	-13,62	85,32	27200,00	308,28
	Z_{66}	16,30	135,17	-60,80	149,11	23400,00	157,58
	Z_{100}	-21,87	187,71	-107,97	217,65	26200,00	121,08
P_z	Z_0	35,38	38,13	60,20	79,56	21200,00	266,46
	Z_{33}	35,38	85,31	-4,74	92,48	22200,00	240,05
	Z_{66}	35,38	132,49	-69,67	153,82	26200,00	170,33
	Z_{100}	35,38	179,67	-134,61	227,28	24800,00	109,12
P_4	Z_0	92,64	30,10	33,56	103,03	22600,00	213,93
	Z_{33}	54,47	82,64	-13,62	99,90	24000,00	238,07
	Z_{66}	54,47	135,17	-60,80	157,90	25600,00	163,79
	Z_{100}	92,64	187,71	-107,97	235,53	25000,00	107,62
T_6	Z_0	128,02	41,60	0,00	134,61	23400,00	173,83
	Z_{33}	66,26	86,47	-24,80	111,73	23600,00	211,23
	Z_{66}	66,26	131,34	-49,61	155,25	24600,00	158,46
	Z_{100}	128,02	176,21	-74,41	230,17	25000,00	108,62
O_1	Z_0	-0,00	-0,00	0,00	0,00	N.R.*	0,00
	Z_{33}	23,59	72,60	-24,80	80,27	N.R.*	300,87
	Z_{66}	23,59	145,20	-49,61	155,25	N.R.*	166,19
	Z_{100}	-0,00	217,81	-74,41	230,17	N.R.*	114,48
O_z	Z_0	35,38	-5,60	0,00	35,83	15600,00	435,44
	Z_{33}	35,38	70,73	-24,80	82,89	22600,00	272,65
	Z_{66}	35,38	147,07	-49,61	159,19	26600,00	167,09
	Z_{100}	35,38	223,41	-74,41	238,12	27200,00	114,23
O_2	Z_0	70,77	-0,00	0,00	70,77	N.R.*	0,00
	Z_{33}	47,18	72,60	-24,80	90,07	N.R.*	256,47
	Z_{66}	47,18	145,20	-49,61	160,53	N.R.*	160,40
	Z_{100}	70,77	217,81	-74,41	240,80	N.R.*	105,90

N.R. = not recordable; *values of ΔV were calculated as average of surrounding ΔVs at each depth

Table S4. Translation of each recording electrodes $E\ (E_x, E_y, E_z)$ on a sphere with origin $T_6(T_{6x}, T_{6y}, T_{6z})$. Distance between each recording point $E\ (E_x, E_y, E_z)$ at each depth Z and $T_6(T_{6x}, T_{6y}, T_{6z})$, with ΔV recorded and EF inferred are reported.

Electrode placement on surface	depth Z	*x (mm)*	*y (mm)*	*z (mm)*	$\overline{T_6E}$	*ΔV (mV)*	*EF (mV/mm)*
Fp_1	Z_0	-128,02	176,21	0,00	217,81	52000,00	238,74
	Z_{33}	-104,43	103,61	-24,80	149,18	49600,00	332,48
	Z_{66}	-104,43	31,01	-49,61	119,70	48000,00	401,00
	Z_{100}	-128,02	-41,60	-74,41	153,81	47600,00	309,47
Fp_z	Z_0	-92,64	181,81	0,00	204,05	39200,00	192,11
	Z_{33}	-92,64	105,47	-24,80	142,56	36800,00	258,15
	Z_{66}	-92,64	29,14	-49,61	109,05	44000,00	403,49
	Z_{100}	-92,64	-47,20	-74,41	127,85	48800,00	381,68
Fp_2	Z_0	-57,25	176,21	0,00	185,28	42300,00	228,31
	Z_{33}	-80,84	103,61	-24,80	133,74	47600,00	355,93
	Z_{66}	-80,84	31,01	-49,61	99,79	47600,00	477,01
	Z_{100}	-57,25	-41,60	-74,41	102,69	44000,00	428,47
F_7	Z_0	-185,28	134,61	0,00	229,01	N.R.*	227,06
	Z_{33}	-123,52	89,74	-24,80	154,68	N.R.*	314,85
	Z_{66}	-123,52	44,87	-49,61	140,47	N.R.*	329,62
	Z_{100}	-185,28	0,00	-74,41	199,66	N.R.*	227,39
F_3	Z_0	-149,89	146,11	33,56	211,99	50400,00	247,76
	Z_{33}	-111,72	93,57	-13,62	146,37	48000,00	337,42
	Z_{66}	-111,72	41,04	-60,80	133,65	46000,00	340,95
	Z_{100}	-149,89	-11,50	-107,97	185,09	45600,00	242,77
F_z	Z_0	-92,64	138,07	60,20	176,83	46000,00	260,13
	Z_{33}	-92,64	90,90	-4,74	129,87	46400,00	357,28
	Z_{66}	-92,64	43,72	-69,67	123,88	46400,00	374,54
	Z_{100}	-92,64	-3,46	-134,61	163,44	46400,00	283,89

F_4	Z_0	-35,38	146,11	33,56	154,03	40800,00	270,78
	Z_{33}	-73,55	93,57	-13,62	119,80	43200,00	364,97
	Z_{66}	-73,55	41,04	-60,80	103,88	45200,00	413,01
	Z_{100}	-35,38	-11,50	-107,97	114,21	46000,00	356,99
F_8	Z_0	-0,00	134,61	0,00	134,61	N.R.*	386,30
	Z_{33}	-61,76	89,74	-24,80	111,73	N.R.*	389,34
	Z_{66}	-61,76	44,87	-49,61	91,04	N.R.*	501,97
	Z_{100}	-0,00	0,00	-74,41	74,41	N.R.*	611,47
T_3	Z_0	-207,15	67,31	0,00	217,81	51200,00	235,07
	Z_{33}	-130,81	67,31	-24,80	149,18	48400,00	324,43
	Z_{66}	-130,81	67,31	-49,61	155,25	45600,00	293,73
	Z_{100}	-207,15	67,31	-74,41	230,17	44000,00	191,17
C_3	Z_0	-163,41	67,31	60,20	186,70	50800,00	272,10
	Z_{33}	-116,23	67,31	-4,74	134,39	48800,00	363,11
	Z_{66}	-116,23	67,31	-69,67	151,31	45600,00	301,38
	Z_{100}	-163,41	67,31	-134,61	222,15	44000,00	199,86
C_z	Z_0	-92,64	67,31	83,19	141,54	44000,00	310,87
	Z_{33}	-92,64	67,31	2,93	114,54	44400,00	387,62
	Z_{66}	-92,64	67,31	-77,34	138,18	44000,00	318,43
	Z_{100}	-92,64	67,31	-157,61	194,81	44400,00	227,91
C_4	Z_0	-21,87	67,31	60,20	92,91	39600,00	426,22
	Z_{33}	-69,05	67,31	-4,74	96,54	41200,00	426,76
	Z_{66}	-69,05	67,31	-69,67	118,96	44000,00	369,86
	Z_{100}	-21,87	67,31	-134,61	152,08	44800,00	294,58
T_4	Z_0	21,87	67,31	0,00	70,77	39200,00	553,91
	Z_{33}	-54,47	67,31	-24,80	90,07	42000,00	466,32
	Z_{66}	-54,47	67,31	-49,61	99,79	46000,00	460,97
	Z_{100}	21,87	67,31	-74,41	102,69	47200,00	459,63
T_5	Z_0	-185,28	0,00	0,00	185,28	N.R.*	0,00
	Z_{33}	-123,52	44,87	-24,80	133,74	N.R.*	351,44
	Z_{66}	-123,52	89,74	-49,61	160,53	N.R.*	277,20
	Z_{100}	-185,28	134,61	-74,41	240,80	N.R.*	181,48

P_3	Z_0	-149,89	-11,50	33,56	154,03	47200,00	313,25
	Z_{33}	-111,72	41,04	-13,62	119,80	46400,00	392,00
	Z_{66}	-111,72	93,57	-60,80	157,90	43600,00	278,96
	Z_{100}	-149,89	146,11	-107,97	235,53	43200,00	185,97
P_z	Z_0	-92,64	-3,46	60,20	110,53	43200,00	390,83
	Z_{33}	-92,64	43,72	-4,74	102,54	43200,00	421,28
	Z_{66}	-92,64	90,90	-69,67	147,30	43200,00	293,27
	Z_{100}	-92,64	138,07	-134,61	213,93	43200,00	201,93
P_4	Z_0	-35,38	-11,50	33,56	50,11	40800,00	643,00
	Z_{33}	-73,55	41,04	-13,62	85,32	40800,00	462,42
	Z_{66}	-73,55	93,57	-60,80	133,65	42400,00	314,26
	Z_{100}	-35,38	146,11	-107,97	185,09	42400,00	225,73
T_6	Z_0	-0,00	0,00	0,00	0,00	N.R.*	0,00
	Z_{33}	-61,76	44,87	-24,80	80,27	N.R.*	518,27
	Z_{66}	-61,76	89,74	-49,61	119,70	N.R.*	366,75
	Z_{100}	-0,00	134,61	-74,41	153,81	N.R.*	289,32
O_1	Z_0	-128,02	-41,60	0,00	134,61	46400,00	344,70
	Z_{33}	-104,43	31,01	-24,80	111,73	44400,00	397,40
	Z_{66}	-104,43	103,61	-49,61	155,25	43200,00	278,27
	Z_{100}	-128,02	176,21	-74,41	230,17	43200,00	187,69
O_z	Z_0	-92,64	-47,20	0,00	103,97	44000,00	423,20
	Z_{33}	-92,64	29,14	-24,80	100,23	43600,00	435,00
	Z_{66}	-92,64	105,47	-49,61	148,89	42800,00	287,46
	Z_{100}	-92,64	181,81	-74,41	217,20	42800,00	197,06
O_2	Z_0	-57,25	-41,60	0,00	70,77	41600,00	587,82
	Z_{33}	-80,84	31,01	-24,80	90,07	42400,00	470,76
	Z_{66}	-80,84	103,61	-49,61	140,47	43200,00	307,55
	Z_{100}	-57,25	176,21	-74,41	199,66	43600,00	218,37

N.R. = not recordable; *values of ΔV were calculated as average of surrounding ΔVs at each depth

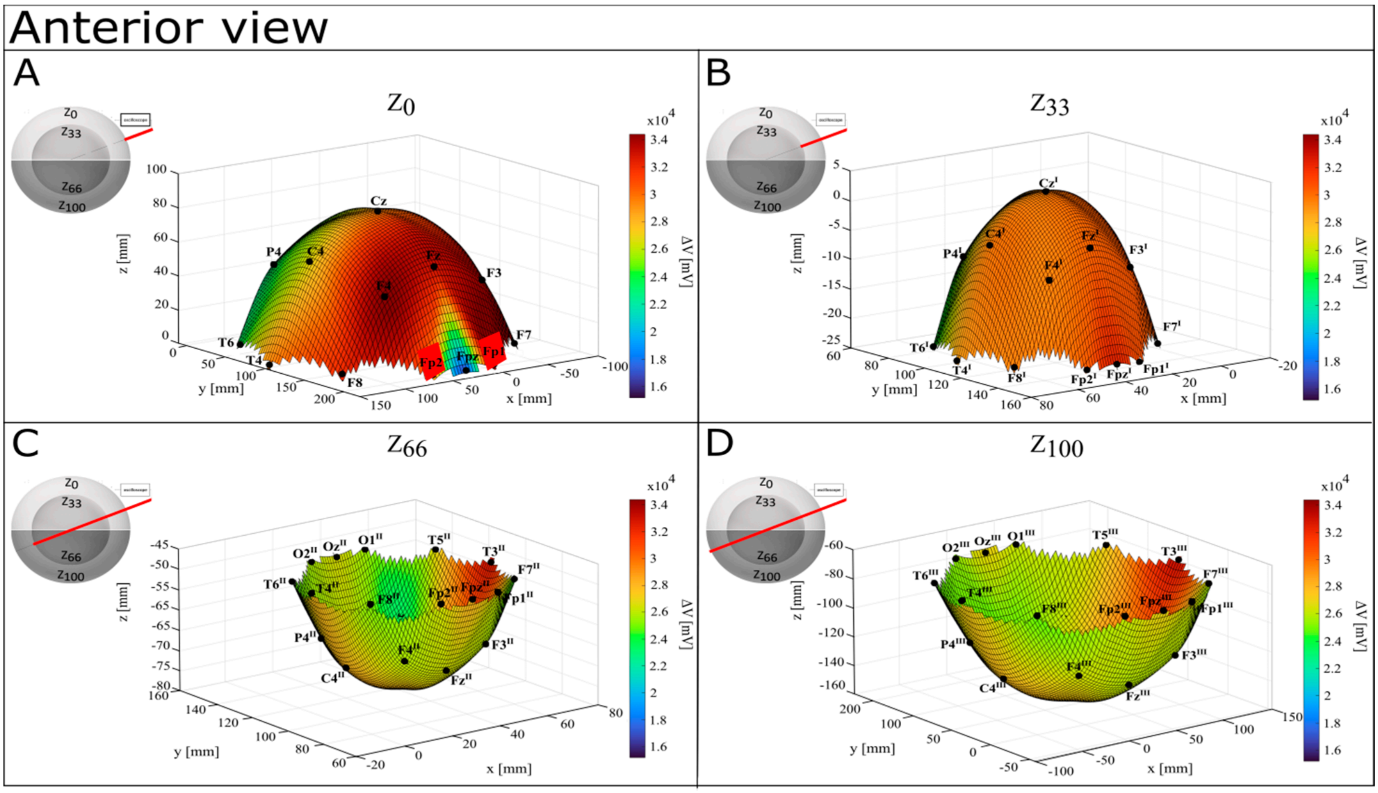

Figure S1. Graphical representation of ΔV distribution in CONDITION B – anterior view. (A) refers to depth Z_0; (B) refers to depth Z_{33}; (C) refers to depth Z_{66}; (D) refers to depth Z_{100}.

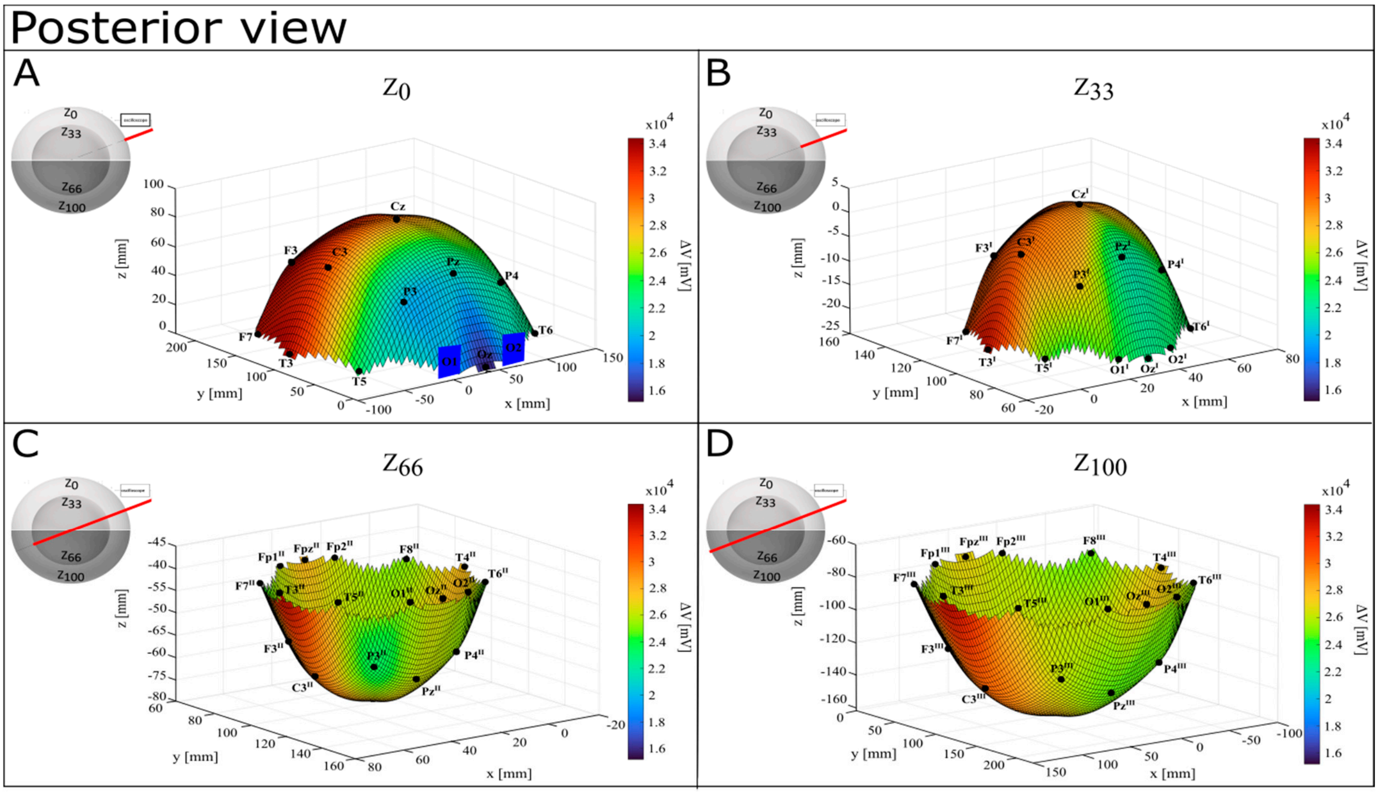

Figure S2. Graphical representation of ΔV distribution in CONDITION B – posterior view. (A) refers to depth Z_0; (B) refers to depth Z_{33}; (C) refers to depth Z_{66}; (D) refers to depth Z_{100}.

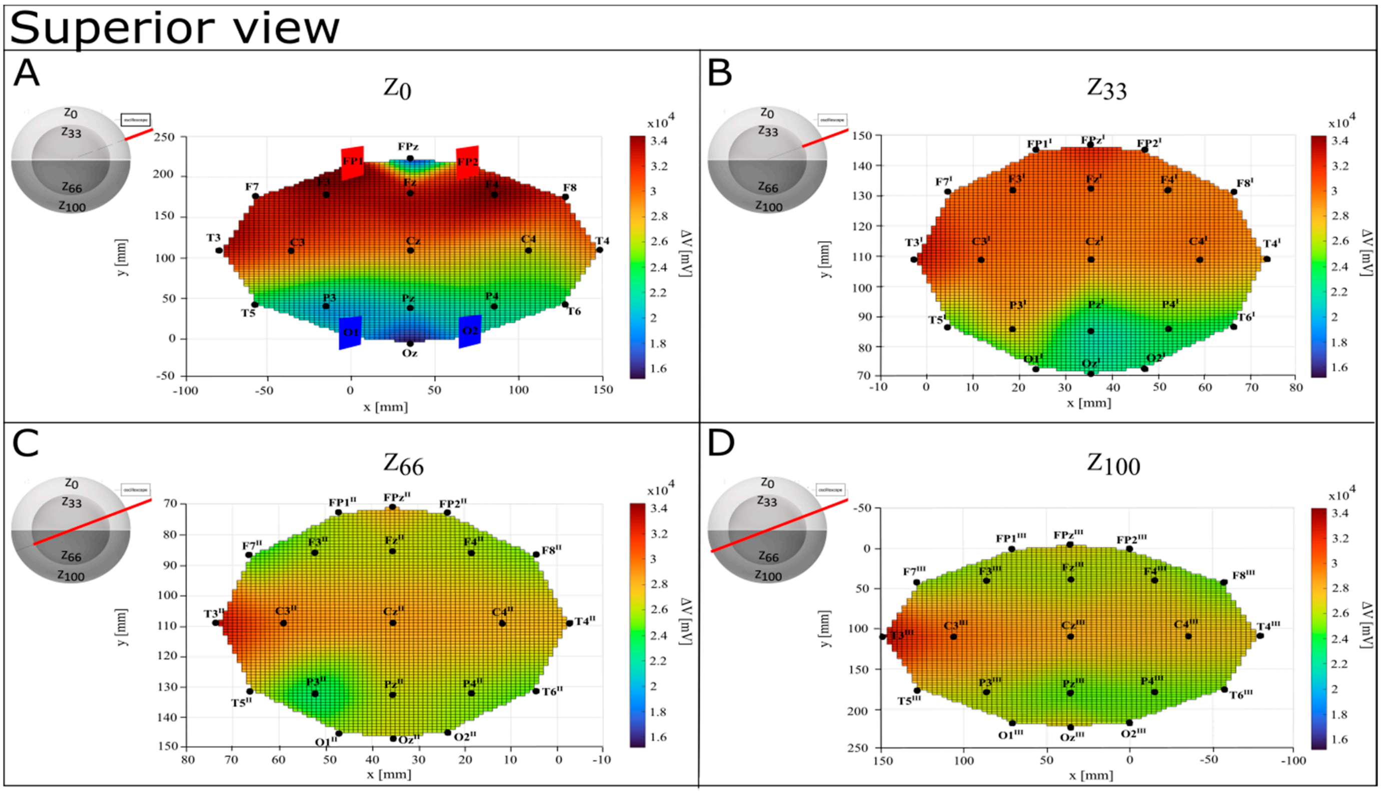

Figure S3. Graphical representation of ΔV distribution in CONDITION B – superior view. (A) refers to depth Z_0; (B) refers to depth Z_{33}; (C) refers to depth Z_{66}; (D) refers to depth Z_{100}.

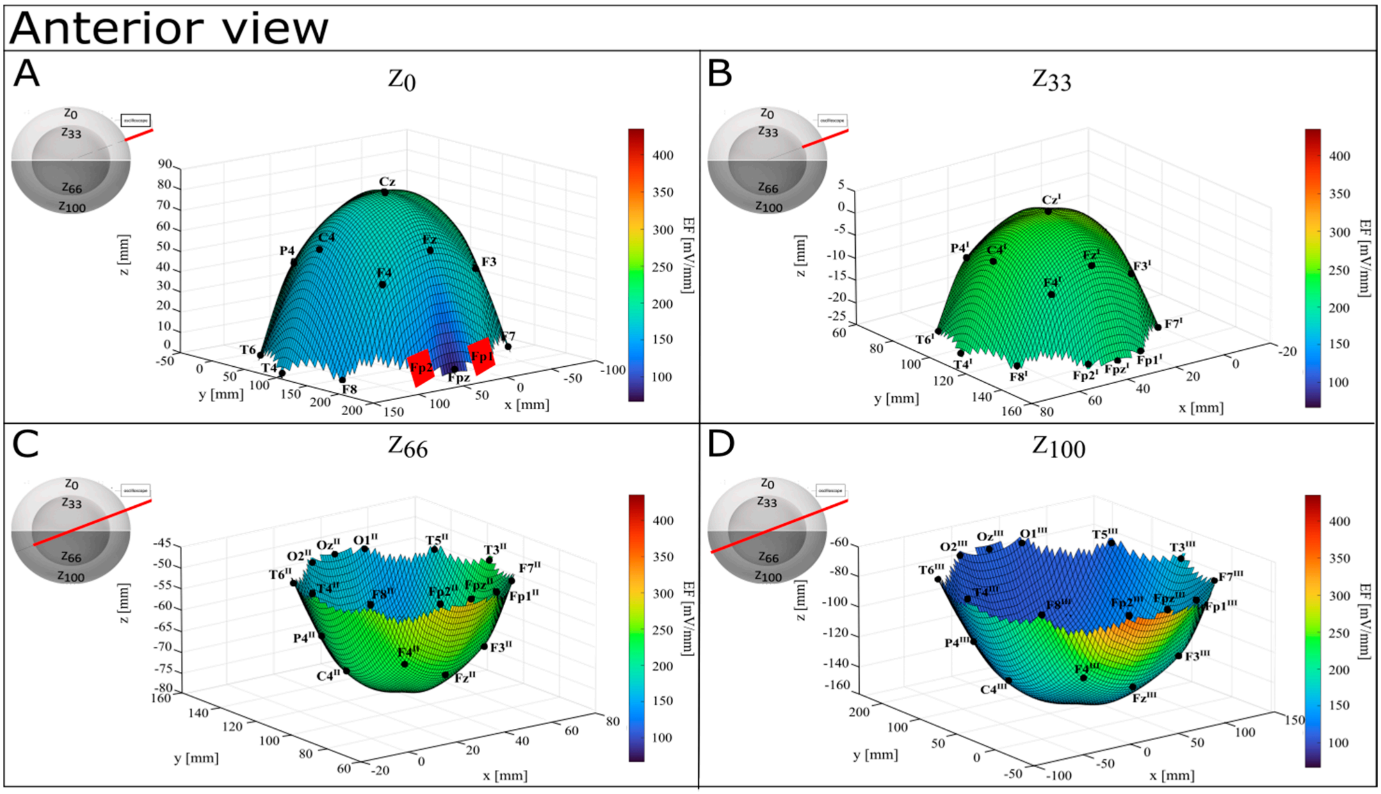

Figure S4. Graphical representation of EF distribution in CONDITION B – anterior view. (A) refers to depth Z_0; (B) refers to depth Z_{33}; (C) refers to depth Z_{66}; (D) refers to depth Z_{100}.

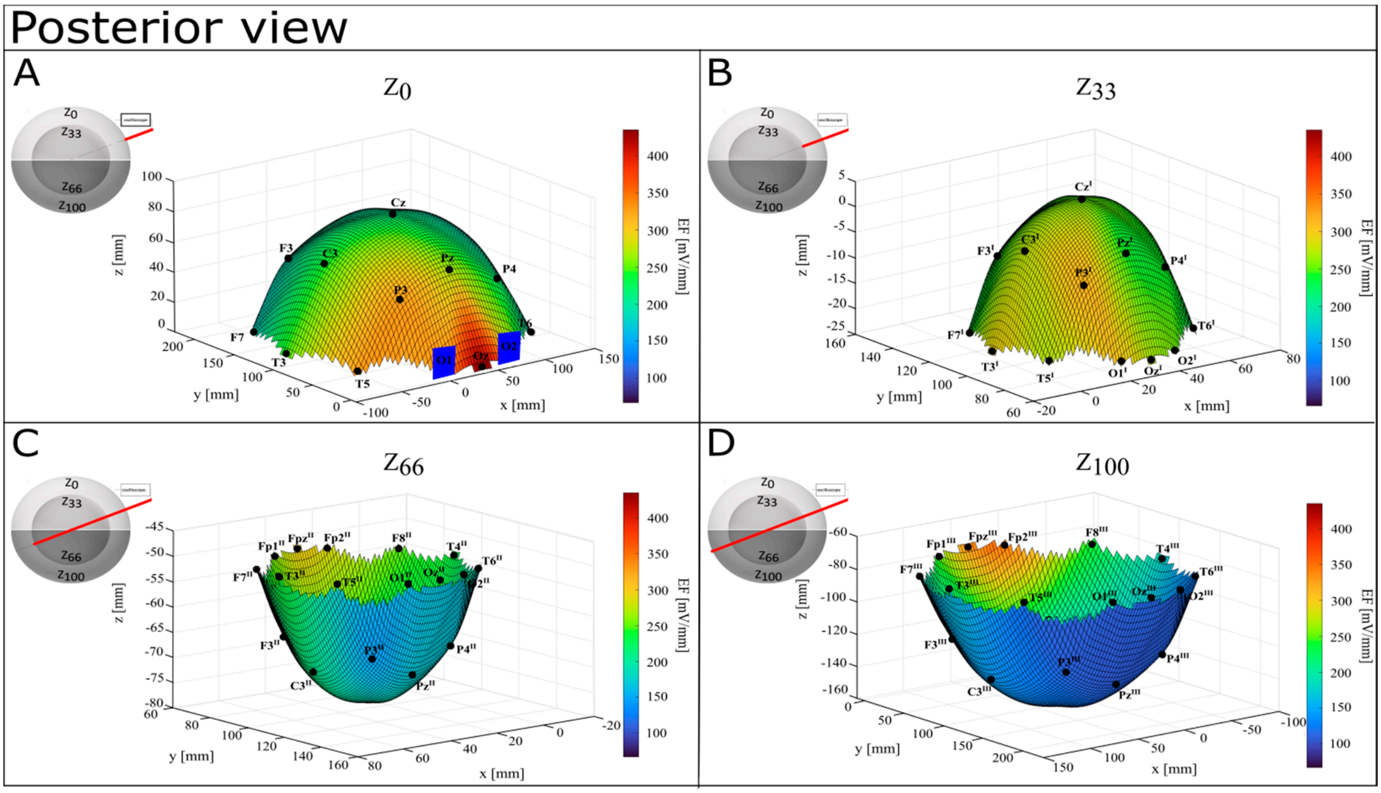

Figure S5. Graphical representation of EF distribution in CONDITION B – posterior view. (A) refers to depth Z_0; (B) refers to depth Z_{33}; (C) refers to depth Z_{66}; (D) refers to depth Z_{100}.

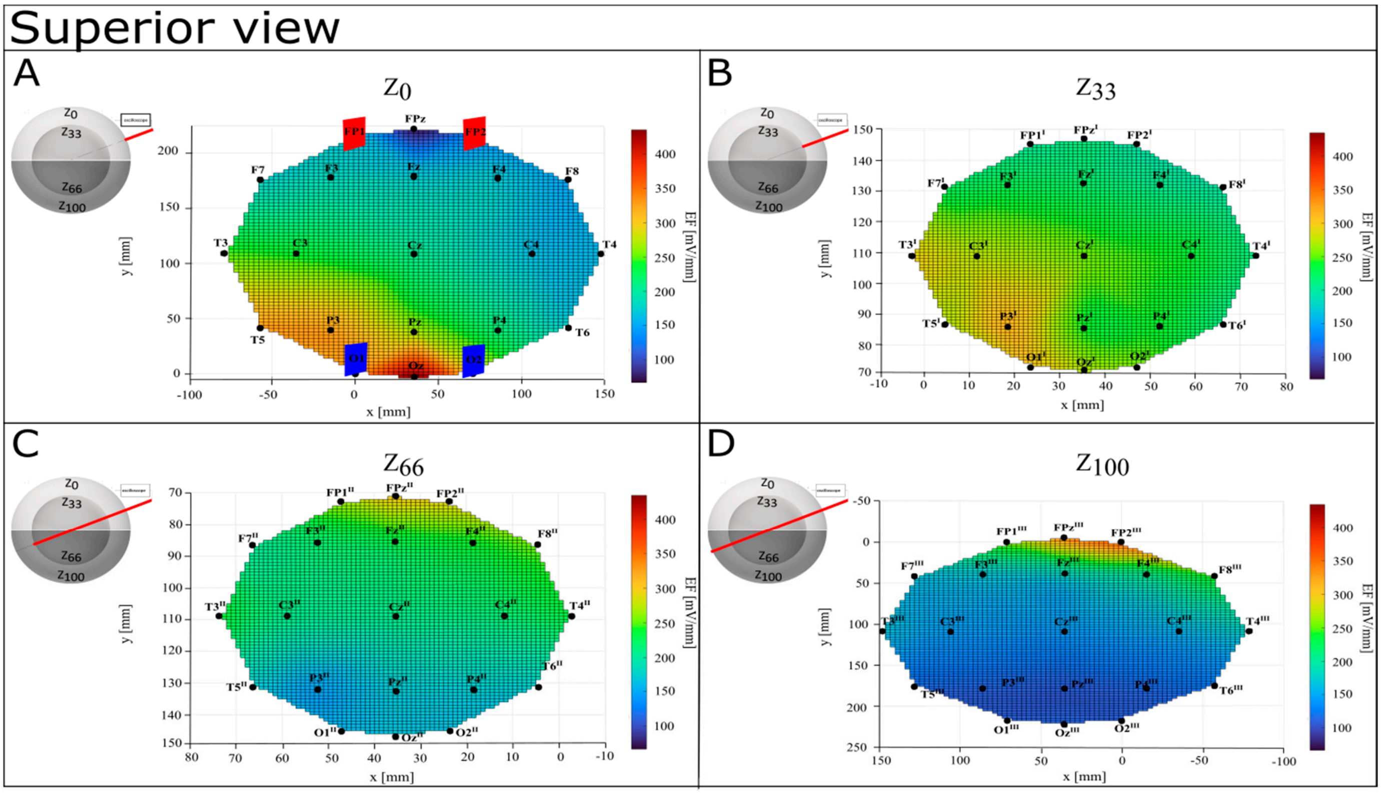

Figure S6. Graphical representation of EF distribution in CONDITION B – superior view. (A) refers to depth Z_0; (B) refers to depth Z_{33}; (C) refers to depth Z_{66}; (D) refers to depth Z_{100}.

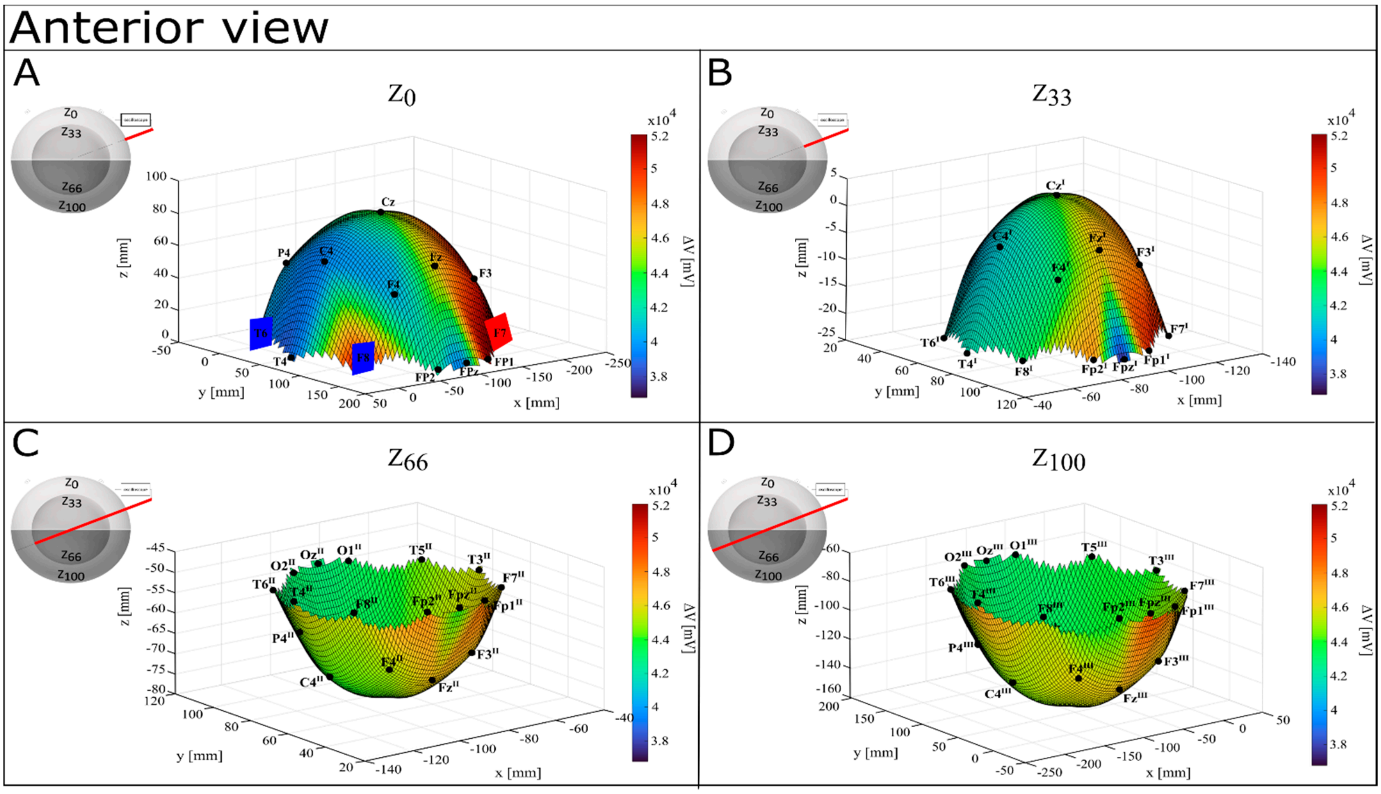

Figure S7. Graphical representation of ΔV distribution in CONDITION C – anterior view. (A) refers to depth Z_0; (B) refers to depth Z_{33}; (C) refers to depth Z_{66}; (D) refers to depth Z_{100}.

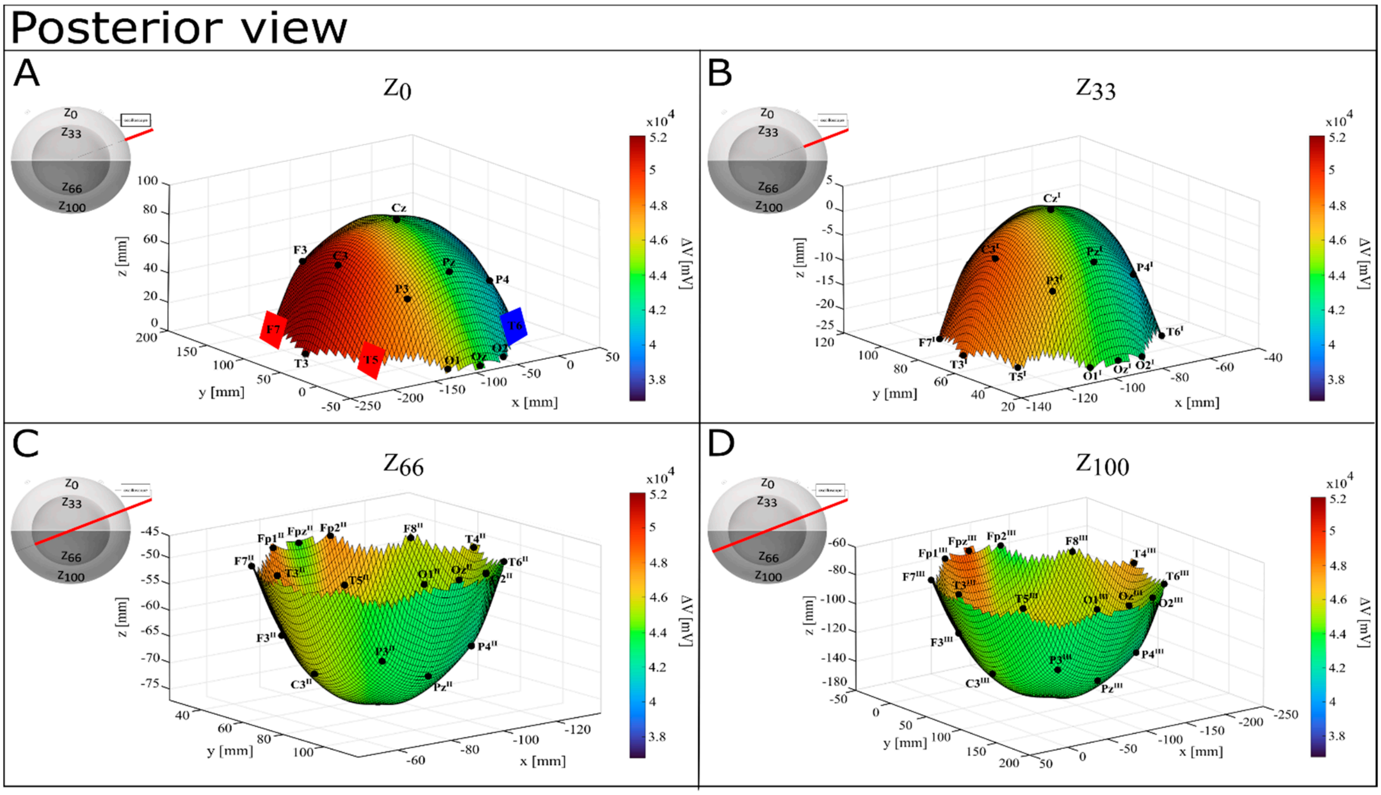

Figure S8. Graphical representation of ΔV distribution in CONDITION C – posterior view. (A) refers to depth Z_0; (B) refers to depth Z_{33}; (C) refers to depth Z_{66}; (D) refers to depth Z_{100}

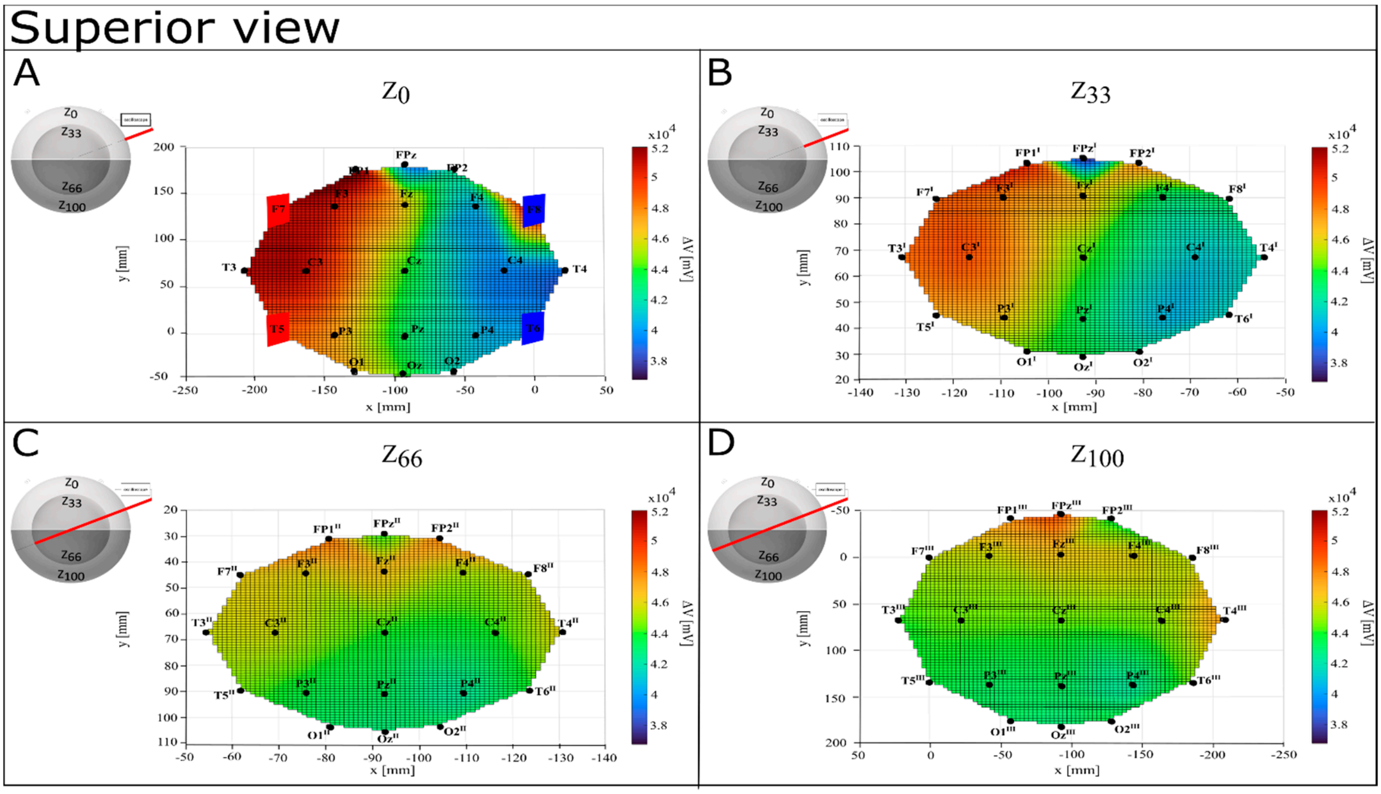

Figure S9. Graphical representation of ΔV distribution in CONDITION C – superior view. (A) refers to depth Z_0; (B) refers to depth Z_{33}; (C) refers to depth Z_{66}; (D) refers to depth Z_{100}.

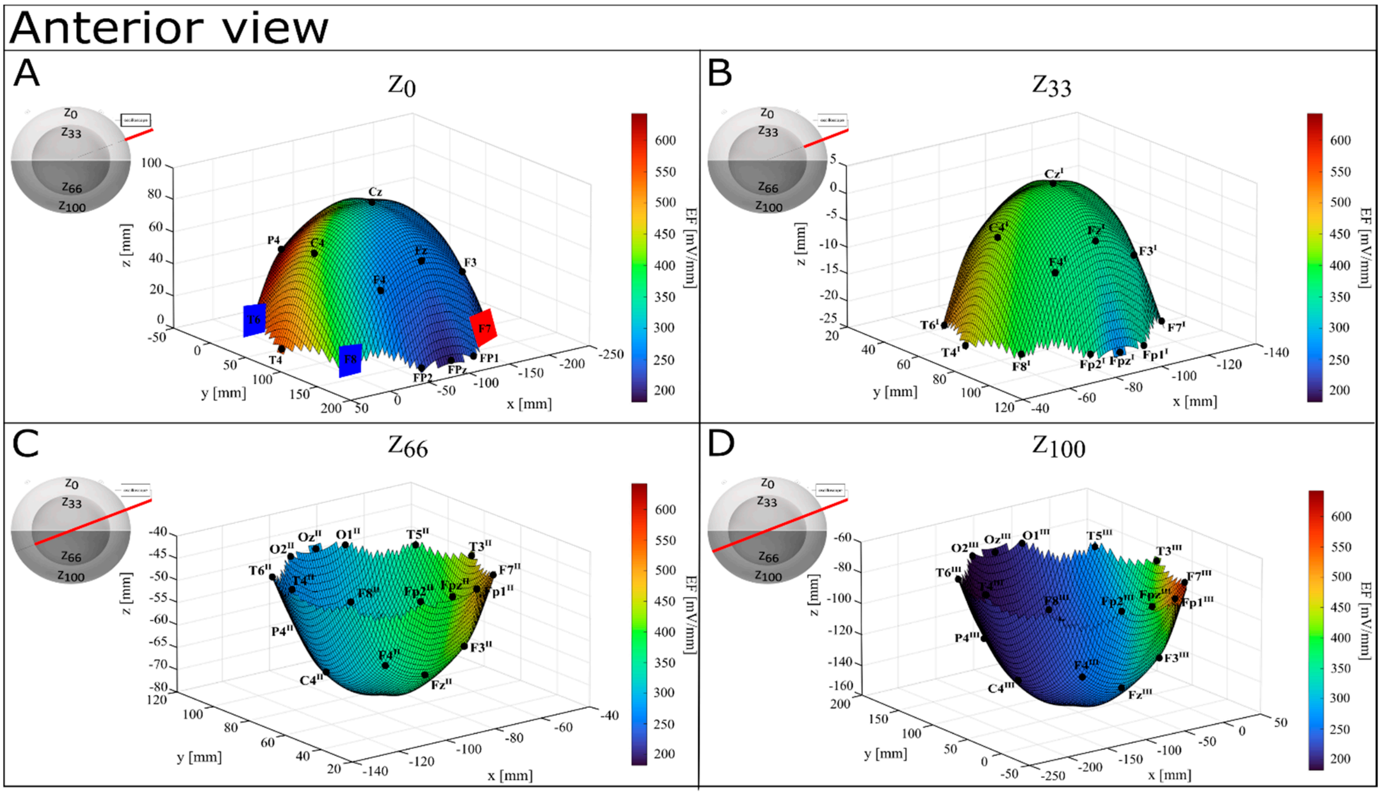

Figure S10. Graphical representation of EF distribution in CONDITION C – anterior view. (A) refers to depth Z_0; (B) refers to depth Z_{33}; (C) refers to depth Z_{66}; (D) refers to depth Z_{100}.

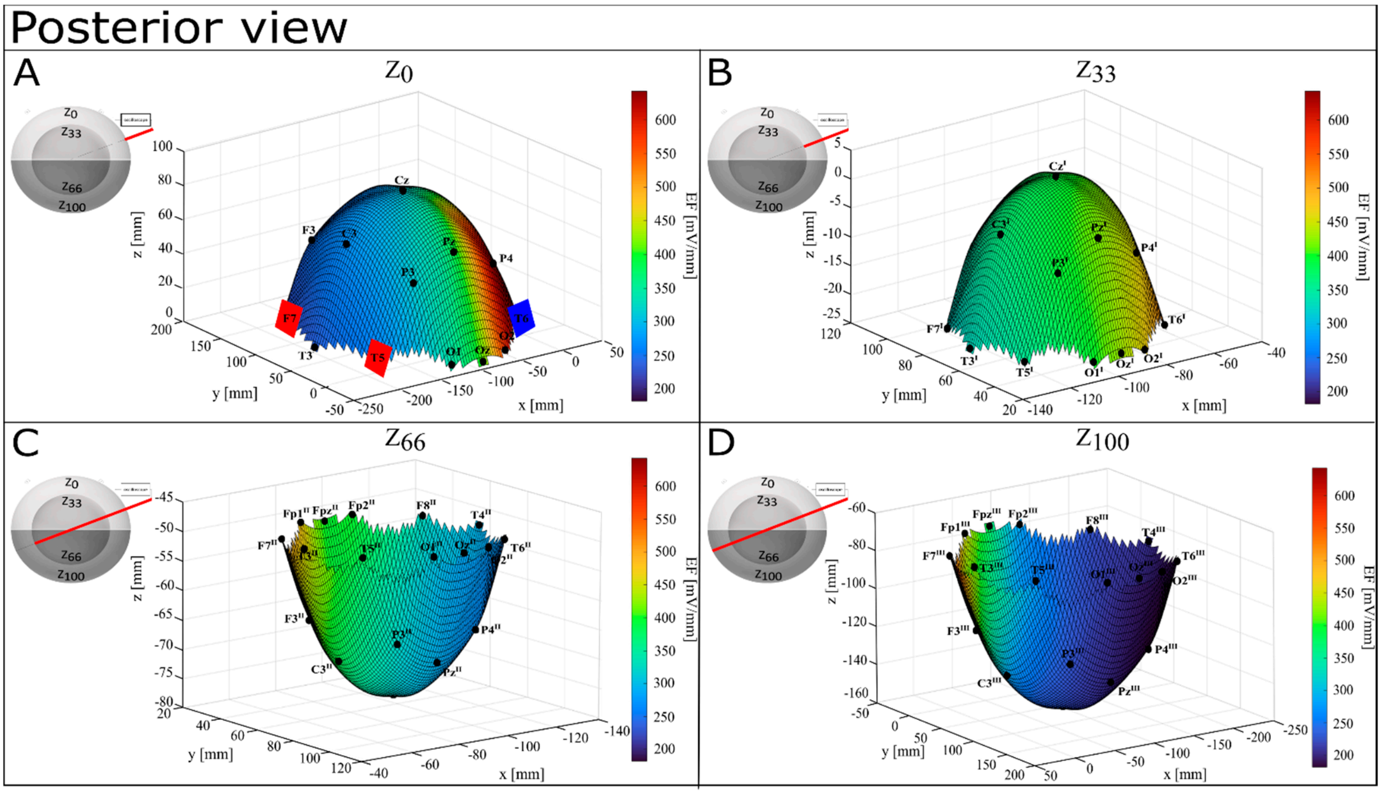

Figure S11. Graphical representation of EF distribution in CONDITION C – posterior view. (A) refers to depth Z_0; (B) refers to depth Z_{33}; (C) refers to depth Z_{66}; (D) refers to depth Z_{100}.

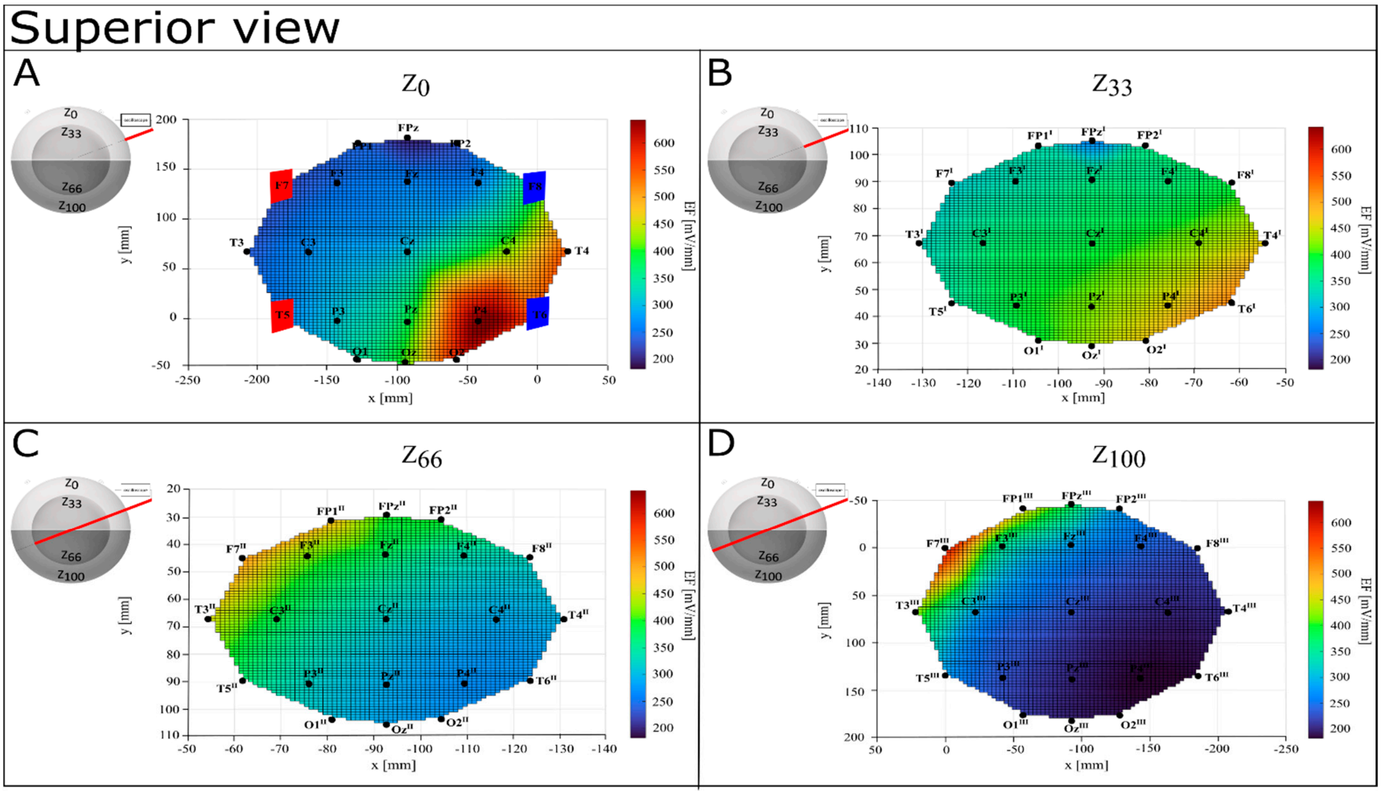

Figure S12. Graphical representation of EF distribution in CONDITION C – superior view. (A) refers to depth Z_0; (B) refers to depth Z_{33}; (C) refers to depth Z_{66}; (D) refers to depth Z_{100}.
